# Models of Formation and Activity of Spring Mounds in the Mechertate-Chrita-Sidi El Hani System, Eastern Tunisia: Implications for the Habitability of Mars

**DOI:** 10.3390/life4030386

**Published:** 2014-08-28

**Authors:** Elhoucine Essefi, Goro Komatsu, Alberto G. Fairén, Marjorie A. Chan, Chokri Yaich

**Affiliations:** 1 National Engineering School of Sfax, Sfax 3038, Tunisia; 2 RU: Sedimentary Dynamics and Environment (DSE), University of Sfax, Sfax 3038, Tunisia; E-Mail: chokriyaich@gmail.com; 3 International Research School of Planetary Sciences, Università d’Annunzio, Pescara 65127, Italy; E-Mail: goro@irsps.unich.it; 4 Department of Astronomy, Cornell University, Ithaca, NY 14853, USA; E-Mail: agfairen@cornell.edu; 5 Centro de Astrobiología, CSIC-INTA, M-108 Km 4, Madrid 28850, Spain; 6 Department of Geology & Geophysics, University of Utah, Salt Lake City, UT 84112, USA; E-Mail: marjorie.chan@utah.edu

**Keywords:** Mechertate-Chrita-Sidi El Hani system, Mars habitability, terrestrial analogs, groundwater upwelling, seepage, tectonic model, hydraulic model, fault spring mounds, artesian spring mounds

## Abstract

Spring mounds on Earth and on Mars could represent optimal niches of life development. If life ever occurred on Mars, ancient spring deposits would be excellent localities to search for morphological or chemical remnants of an ancient biosphere. In this work, we investigate models of formation and activity of well-exposed spring mounds in the Mechertate-Chrita-Sidi El Hani (MCSH) system, eastern Tunisia. We then use these models to explore possible spring mound formation on Mars. In the MCSH system, the genesis of the spring mounds is a direct consequence of groundwater upwelling, triggered by tectonics and/or hydraulics. As they are oriented preferentially along faults, they can be considered as fault spring mounds, implying a tectonic influence in their formation process. However, the hydraulic pressure generated by the convergence of aquifers towards the surface of the system also allows consideration of an origin as artesian spring mounds. In the case of the MCSH system, our geologic data presented here show that both models are valid, and we propose a combined hydro-tectonic model as the likely formation mechanism of artesian-fault spring mounds. During their evolution from the *embryonic* (early) to the *islet* (“island”) stages, spring mounds are also shaped by eolian accumulations and induration processes. Similarly, spring mounds have been suggested to be relatively common in certain provinces on the Martian surface, but their mode of formation is still a matter of debate. We propose that the tectonic, hydraulic, and combined hydro-tectonic models describing the spring mounds at MCSH could be relevant as Martian analogs because: (i) the Martian subsurface may be over pressured, potentially expelling mineral-enriched waters as spring mounds on the surface; (ii) the Martian subsurface may be fractured, causing alignment of the spring mounds in preferential orientations; and (iii) indurated eolian sedimentation and erosional remnants are common features on Mars. The spring mounds further bear diagnostic mineralogic and magnetic properties, in comparison with their immediate surroundings. Consequently, remote sensing techniques can be very useful to identify similar spring mounds on Mars. The mechanisms (tectonic and/or hydraulic) of formation and evolution of spring mounds at the MCSH system are suitable for the proliferation and protection of life respectively. Similarly, life or its resulting biomarkers on Mars may have been protected or preserved under the spring mounds.

## 1. Introduction

Terrestrial analogs to Mars are the subject of great attention due to the similar geological histories of the two planets [1,2,3] and due to the intense exploration of Mars that has occurred over the last decade [4]. This similarity may be extended from the simple analogy between geomorphologic features on the two planets to the identification of identical formation models and geological processes. The Martian subsurface could be more dynamic, geologically active, and having more hospitable conditions for life than its surface [5]. However, the direct study of the deeper Mars subsurface is currently out of reach due to major technical and financial challenges. Accordingly, indirect studies of the Martian subsurface through comparison with analog terrestrial sites are the only feasible alternative today.

Worldwide, a number of sites have been studied for their application to understand geological and potential biological processes on Mars (e.g., [6,7,8]). To name but a few, the Channeled Scablands (e.g., [9,10]), central Australia (e.g., [11]), Sahara Desert (e.g., [12,13]), Tunisia (e.g., [14,15,16,17]), the Dead Sea (e.g., [18]), and the Arctic and Antarctica [2,19,20] are sites sharing similarities with Mars. Analogies of these terrestrial lands to Mars have been proposed from several viewpoints, including sedimentological (e.g., [21,22,23,24,25,26,27]), geochemical [22,23,28], mineralogical (e.g., [29,30]), hydrogeological (e.g., [25,30,31,32,33,34,35]) and biological (e.g., [29,31,36,37]).

In the analysis of terrestrial analogs, saline environments in their widest context have received special attention. The most general definition of a saline environment encompasses the depressions and the surrounding hydrological and hydrogeological watersheds. Depressions include sediment and water, showing topographical, geochemical, putative biological, and sedimentological similarities with Martian deposits, such as the sebkha sediments of the Burns formation [21,27]. Hydrological watersheds include eolian and hydraulic deposits, showing geomorphologic, stratigraphic, and sedimentologic similarities with Martian landscapes, such as the eolian sediments of the Burns formation [21,27]. Hydrogeological watersheds may converge toward discharge playa surfaces and cause the formation of springs and spring mounds (e.g., [35,38,39,40]). On Mars, an analogous process of convergence of fluids could be in the origin of spring mounds (e.g., [35,41,42,43]), conical features [44], and mud volcanoes (e.g., [45]). Occurring on the surface, these features show an exceptional scientific interest, because, (i) they extrude subsurface materials to the surface, which are therefore readily accessible for analysis; and (ii) subsurface materials would have been more probable ecological niches for the development of extremophile organisms [41], as they or their remains could have been protected from the harsh conditions that have dominated the surface of Mars during at least the last 3.5 Ga. These mounded spring deposits are a unique subset of spring sediments, which develop through the accumulation of suspended sediment, peat, eolian material and groundwater precipitates in areas of direct discharge [46]. In the few modern environments where they are found, artesian spring mounds are often evaporative systems that allow for evaporites (e.g., carbonate) precipitation near active spring vents (e.g., [47,48]). The precipitates that result from this evaporative process contribute to the overall development of the mound form, while precipitate mineralogy is controlled by groundwater chemistry [49].

The apparent similarity between terrestrial analogs and Martian systems likely reflects parallel modes of formation and geological processes. Here we argue that the Mechertate-Chrita-Sidi El Hani (MCSH) system in eastern Tunisia may be considered as a potential analog to Mars due to the presence of specific geologic features on the surface of its depressions, such as spring mounds [35], which appear to be similar to those observed on the surface of Mars [41]. The enhancement of spring mounds formation within MCSH is still an enigma. On the one hand, previous hydrogeological and geochemical works [38,39,40,50] advocate the hydraulic initiation of these features. On the other hand, more field expeditions and tectonic analyses [35] suggest that the tectonic origin is worth to be defended. In this paper, after a multi-disciplinary investigation of the terrestrial analog of MCSH, we discuss different geological models that may indicate the existence of surface-subsurface connectivity leading to the formation of spring mounds in similar terrestrial environments and Martian systems. We will also infer the hydraulic and geodynamic conditions at the subsurface of Mars, which are likely favorable for life development.

## 2. Study Areas

**Figure 1:**
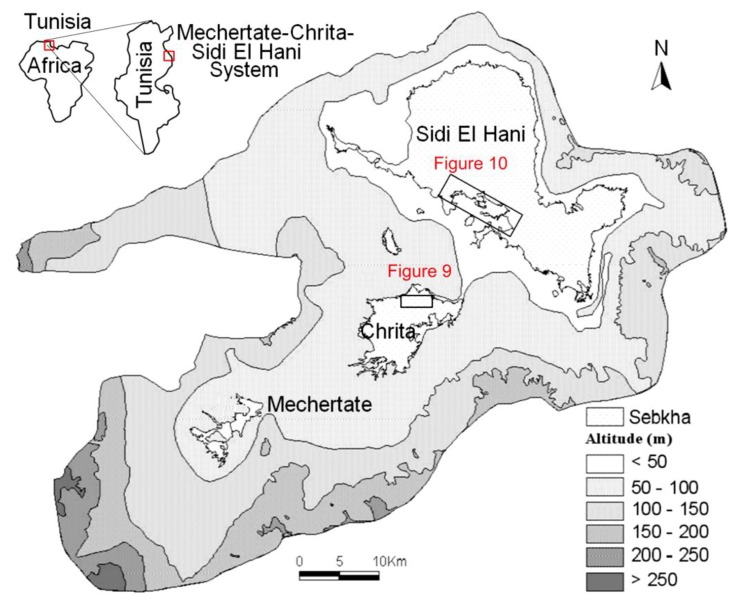
Geographical location and topography of the Mechertate-Chrita-Sidi El Hani system [53]: the rectangles within depressions of Chrita and Sidi El Hani indicate positions of Figure 9 and Figure 10 respectively.

The word “systema” originated from the Greek means an organized set. Many authors tried to define the concept used in different scientific disciplines [51,52]. On Earth, the term “endorheic system” was used as the hierarchical combination between endorheic basins [52]. Previous geomorphologic studies (e.g., [53,54]) defined the endorheic system of Mechertate-Chrita-Sidi El Hani (MCSH) (Figure 1). In addition, Essefi [42] integrated the tectonic settings and the hydrogeological context in the multidisciplinary definition of this system. Similarly, many Martian sites satisfy the conditions of surface-subsurface connectivity. To name but a few, Meridiani Planum [55], Gale crater [31], and Vernal Crater [25] are zones with at least groundwater influence controlling the sedimentation and setting of specific features such as springs and putative spring mounds. In this paper, due to their apparent similarity [35], the putative spring mounds [25] at Vernal Crater are compared to the spring mounds at the system of Mechertate-Chrita-Sidi El Hani (MCSH).

### 2.1. The System of Mechertate-Chrita-Sidi El Hani (MCSH)

The system of Mechertate-Chrita-Sidi El Hani (MCSH) consists of three interconnected sub-systems: Mechertate, Chrita and Sidi El Hani (Figure 1). The sebkha of Mechertate is located in the upstream of the system. Neither satellite images nor field works allowed researchers [38,53] to find spring mounds. The Chrita saline lake is located in a mid-way between Mechertate playa and Sidi El Hani discharge playa. Satellite images show spring mounds on its surface (Figure 9). The sebkha Sidi El Hani may be considered as the terminal area of the system since it collects all the exceeding water and sediment. Though it has historically been treated as a single unit (e.g., [56]), the sebkha Sidi El Hani as such is actually made up of three communicated playas (Figure 1): from north to south, the playas ofSidi El Hani (*sensu stricto*),Souassi,and Dkhila. The three components of the sebkha Sidi El Hani have different orientations. Sidi El Hani (*sensu stricto*) and Dkhila playas are oriented N170; the playa of Souassiis orientedN90. These shapes were inherited from the tectonic phases that controlled the genesis and formation of saline depressions in eastern Tunisia [56].

Hydrologically and hydrogeologically, the Sidi El Hani discharge playa represents the basal part of the endorheic system [38,39,40,53], in which the Kairouan, Souassi and Zarmdine aquifers converge after leaching subsurface domes of salt and/or transporting salty water also located in the subsurface of the system [38,39,50]. This convergence explains the existence of huge quantities of salt (halite) and brine within the discharge playa [38,50,57]. Being the downstream of the system, this discharge playa is rich with spring mounds, which were noticed by satellite images and during field works [38,53]. Small islets also occur within Chrita and Sidi El Hani depressions (Figure 4b); they radically differ from their surroundings. They are covered with a thick layer of eolian sediment and their internal sediment tends to be more muddy [38]. They may be originally initiated as spring mounds. Then, the intensive eolian sedimentation increases their sizes toward their current forms as distinctive bodies within depressions.

### 2.2. Vernal Crater: A Typical Martian Site for Mounds Formation

Vernal Crater is a 55 km diameter located at 6°N, 355.5°E, in southwestern Arabia Terra. It is one of the few equatorial regions on Mars with high abundance of near-surface hydrogen [25]. This abundance argued the presence of shallow ice or hydrated minerals [58,59]. Vernal Crater is, a Noachian impact structure that exhibits layered sediments, potential remnants of fluvio-lacustrine activity, and indications of eolian processes (e.g., [25,41]).

At Vernal Crater, we focus on the outcrop of putative spring mounds [25]. These features are the result of subsurface fluid migration. The outcrop fills at Vernal Crater slopes uniformly from the northwest rim down to the level of the springs and provides a potential hydraulic head advocating hence the hydraulic origin of spring mounds. Such migration is likely to occur along bedding planes, faults/fractures, or porous units in Vernal Crater’s fill. Faults, fractures, and porous carrier beds perhaps played a role in the subsurface movement of fluids at Vernal Crater and that flow could be artesian and/or thermal.

## 3. Methods

Due to the complexity of the geologic context of the MCSH system [38,53], we have performed a multidisciplinary and multi-scale approach. The scale of our study varied from a few meters to a few kilometers: correlations between vibrocore drills (drilled for hydrogeologic purposes) and geodynamic interpretations stretched over kilometers, while spring mound examinations stretched on the scale of few meters. This study consists of tectonic and hydrogeologic investigations of the system as a whole. The focus was meant to be on a multi-disciplinary investigation of spring mounds present in the Chrita saline lake and the Sidi El Hani discharge playa. Subsequently, collecting the dispersed jigsaw puzzle of different approaches, we propose models of spring mound formation and activity in the system and on Mars.

### 3.1. Tectonic Framework of the MCSH System

**Figure 2:**
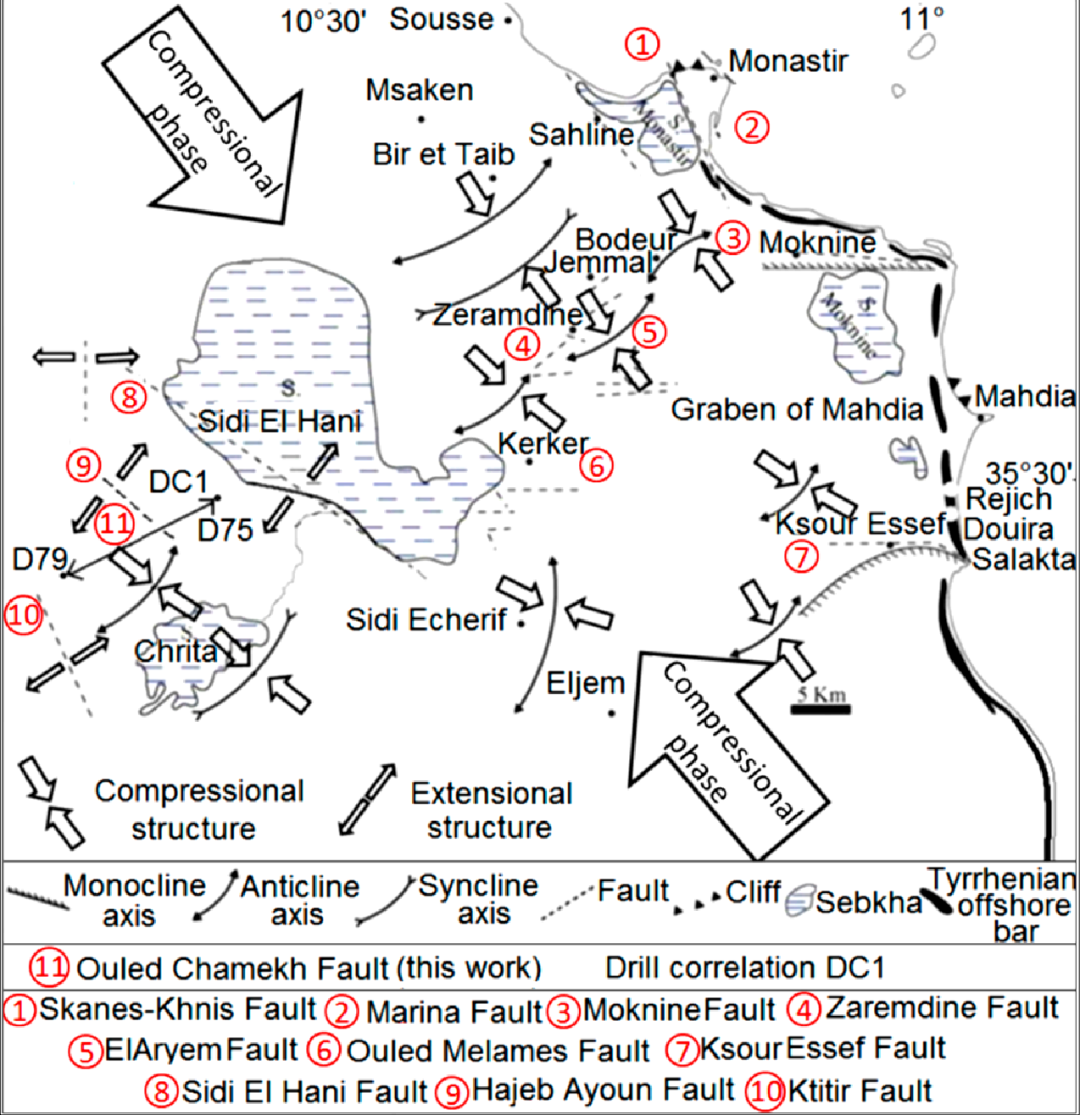
Tectonic map of the Sahel area showing the past tectonic activity of the region: relation between extensional and compressional structures and the compressional phase originated from Africa and Eurasia plate movement. Mechertate-Chrita-Sidi El Hani: site of drills correlation (DC1) (modified and reinterpreted from Ghribi [62]): NW-SE is the major tectonic alignment, whereas NE-SW to E-W orientation represents the minor tectonic alignment.

The tectonic study was based on a correlation between the sedimentary contents of two vibrocore drills (raw data from the Ministry of Agriculture, Tunisia) obtained from the vicinity of the Sidi El Hani discharge playa (Figure 2; DC1: Drills Correlation between D79 and D75). This correlation showed a syn-sedimentary fault at the level of Ouled Chamekh. Similar faults such as the fault of Sidi El Hani (Figure 2) might have given birth to spring mounds within the system. This work also combined the geodynamic map previously discussed by Ben Ayed and Zargouni [60] and recently modified by Zouaghi *et al.* [61] and the tectonic alignment of islets at the Sidi El Hani discharge playa [35] to show the setting of these islets within the geodynamic context of African and Eurasian plates convergence.

### 3.2. Hydrogeologic Framework of the MCSH System

For the hydrogeological study, the hydrogeological mapping of the system (Figure 5) was carried out based on 50 vibrocore drills within the hydrogeological watershed of the system (raw data from the Ministry of Agriculture, Tunisia), four cores within the Chrita saline lake, and 9 cores within the Sidi El Hani discharge playa. Combined with the hydrodynamics of the hydrogeological system within the Kairouan aquifer-Sidi El Hani discharge playa recently discussed by Essefi [38], this map allowed the elaboration of a model relating depressions of the system (Mechertate and Chrita saline lakes and Sidi El Hani discharge playa) and the Kairouan aquifer. The model may give an explanation to the springs of water and spring mounds in the system.

### 3.3. Spring Mound Investigations

For the study of the spring mounds *senso stricto*, we also followed a multi-disciplinary approach to understand the mechanism of their formation and activity. This approach encompassed tectono-topographic, sedimentological, hydrogeological, mineralogical, and magnetic studies.

#### 3.3.1. Tectono-Topographic Study: Spring Mound Morphology

In the tectono-topographic investigation, Google Earth images (2011) (Figure 9) were investigated at different scales (132 m, Figure 6a; 267 m, Figure 6b; 79 m, Figure 9c) to identify spring mounds in the Chrita saline lake. At the Sidi El Hani discharge playa, spring mounds wffigurere also identified (Figure 10) at metric scales (213 m, Figure 7a; 282 m, Figure 7b; and 85 m, Figure 10c). Tectonically, alignments of spring mounds along preferential orientations were interpreted to infer the tectonic activity within both depressions and to link them with the global geodynamics of the region.

#### 3.3.2. Spring Mound Sedimentology

Previous sedimentologic investigations of spring mounds focused on field investigations and the study of the descriptive grain size distribution [63]. Field investigations were organized to explore these structures on the ground. Spring mounds were photographed and their dimensions were measured by a folding rule. For the sedimentological study, we cored within the sedimentary content of these spring mounds in order to identify the sedimentary facies along cores and to correlate between different levels. Coring is an efficient tool for spring mounds investigation on earth as well as on Mars (e.g., [26]). In this study, coring was carried out by the penetration of an empty tube (6.3 cm diameter). Penetration was by rotation and slight push on sediment in order to avoid the compression of sediment. In laboratory, the tube was divided into two equal halves in order to visualize the sedimentary facies (Figure 12a, Figure 14a, Figure 16a). Along all the cores, we used the Visual Core Description (VCD) (e.g., [64]) to identify facies based on their colors and visual characteristics. From every spring mound, one core was investigated in terms of genetic grain size distribution. Wet process analyses were carried out by the FRITSCH laser grain size analyzer. This investigation distinguished between the eolian, the geochemical, and the hydraulic sedimentations based on modes of the grain size distribution [54,64,65,66] Sun *et al.* [65] considered the fraction centered around 6 μm as fine eolian component and the fraction centered around 60 μm as coarse eolian component. Whereas the coarse hydraulic component is centered around 380 μm and the fine hydraulic fraction is centered around 1 μm. Based on their cumulative curves, Cailleux and Tricart [66] distinguished between 23 types of sedimentation: six estuary and deltaic, seven marine, two glacial, three eolian, and five fluvial types. More significance has long been attributed to the shapes of the cumulative grain-size curves rather than distribution curves of sediments (e.g., [66,67]). However, in the details of their form, grain-size distribution curves are more telling than cumulative curves [68]. The shape of the distribution curve displays various “features” (F) characteristic of the dispersed sediment. Features are classified according to their frequencies. The primary (M) and secondary (m) modes have the highest frequencies. Shoulder-like segments (S) are of a lower strength than the primary and secondary modes. Particular features are absent (A) from some samples and unrealized (occluded, O) in others. Added to the traditional sand/silt/clay subdivision used in the literature, Manté *et al.* [69] coined the term colloids as the fraction between 0.063 µm and 1 µm. This fraction is of a geochemical origin. Grain-size components of eolian deposits depend on the nature of winds (*i.e.*, high- and low-altitude air flows and near-ground winds) and transport distances (long or short distance) [65,70,71]. Based on the method of features of Allen and Haslett [68], the descriptive classification of Flemming [72], and the three reference cumulative curves of eolian types (their transformation toward frequency curves) discussed by Cailleux and Tricart [66], Essefi *et al.* [54] distinguished between three types of eolian sediments. First, the eolian sand could be transported by strong wind. Its most important features are the mode at 500 µm and the two shoulders at 250 µm and 1600 µm. Consequently, this eolian sediment is classified according to sand/silt/clay diagram of Flemming [72] as sand. Second, the slightly silty eolian sand [72] could be transported by a moderate wind. The most important features are the mode at 315 µm and the two shoulders at 200 µm and 800 µm. Third, the silty eolian sand [72] could be transported by calm wind. The most apparent futures are the mode at 160 µm and the two shoulders at 250 µm and 1000 µm. To conclude, the fractions centered around 6 and 60 µm [65]; 160, 315, 500 µm [54] mark the eolian component. The hydraulic component is marked by the fractions 1 µm and 380 µm [65]. The geochemical fraction is marked by colloids, which are smaller than 1 µm [69].

#### 3.3.3. Spring Mound Hydrogeology

For the hydrogeologic study of the spring mounds, aquifer levels encountered during coring allowed the elaboration of hydrogeologic maps. The knowledge of water table allows the identification of water flows within active and inactive spring mounds.

#### 3.3.4. Spring Mound Magnetic Properties

During the last few years, magnetic susceptibility mapping has become in terrestrial geology an established method to study the spatial distribution of different soils. It has been used for investigations around power plants [73,74], iron industry and mining areas [75,76,77,78], urban environments [78] and roads [79]. It has also proved to be useful for studying the influence of atmospheric processes on distribution and deposition of air pollutants [80,81] and for discriminating different soil-contamination sources [82]. The laboratory experiment results [83] showed a variation of measured magnetic susceptibility under different degrees of moisture, indicating mainly the influence from the diamagnetic contribution of the water volume. The magnetic susceptibility could be used to identify areas of deposition or detachment. The magnetic susceptibility would be increased or reduced depending on whether deposition or detachment occurs [84]. To distinguish the sedimentary content of these spring mounds from their surroundings, magnetic properties of sediment of a spring mound surface (Figure 16b; MS11, MS12, MS13, MS14) and its surrounding (Figure 16b; MS21, MS22, MS23, MS24) in the Sidi El Hani discharge playa were investigated. The low and high frequency magnetic susceptibility (MS) were measured by the Bartington MS2B probe in the laboratory of Sedimentary Dynamics and Environment, National engineering School of Sfax, at frequencies of 0.47 kHz and 4.7 kHz. Samples were packed into 10 cm^3^ cylindrical perspex pots for MS analysis. The results were expressed as mass susceptibility XLF and XHF, and the corresponding frequency-dependent susceptibility was calculated as difference percentage: XFD = XLF − XHF/XLF × 100%.

#### 3.3.5. Spring Mound Mineralogy

Because all minerals diffract X-rays in a distinguishable pattern, scientists use the information from X-ray diffraction to identify the crystalline structure of materials on earth (e.g., [85,86,87] and on Mars (e.g., The Chemistry and Mineralogy instrument (CheMin) on Curiosity). Analogically to our investigations in ancient (rich with organic matter) [87] and relatively recent (with groundwater influence) [85] terrestrial sites, using CheMin, scientists will be able to further study the role of water in Martian mineralogy and the potential organo-mineral complex originating from primitive life on Mars. In addition, combined with magnetic investigation [88], the study of Martian mineralogy may explain the link between the self magnetization of Martian crust [89] and magnetic motifs [90] The mineralogical study of spring mounds also provides with models of formation and functioning [63]. Two samples (Figure 18; H2-4; H48-50) were selected from the top and the bottom of a core from an active spring mound (Figure 16; G1). The mineralogical composition of the bulk rock of the two samples was determined by X-Ray Diffraction (XRD). The used diffractometer is Philips X-PERT with a Cu anticathode (Ka). The recording and the digital processing of the data are carried out using the software X’ PERT HighScore Plus®.

The models of formation and activity of spring mounds in the MCSH system and on Mars was hypothesized based on a cross interpretation of results of the tectonic and hydrogeologic studies of the system on one hand, and the tectono-topographic, sedimentological, and hydrogeological results of spring mound investigations on the other.

## 4. Results

Spring mound formation and activity are controlled both by past and current tectonic and geodynamic settings [91], and by the current hydrogeological context [35]. The evolution of spring mounds is controlled by a wet aeolian sedimentation.

### 4.1. Past and Current Tectonics and the Geodynamic Context of the MCSH System

Though structures of the previous tectonic activities in the Sahel area are covered by a thick Plio-Quaternary series, geologic and geophysical studies (e.g., [56,61,62,92,93,94,95]) provided a wealth of data about the deep tectonic structures and the salt tectonics that were enhanced by the intrusion of Triassic domes (e.g., [93,94]). The geodynamic inheritance in the subsurface of this region is still controlling the geology of its surface until today [91]. For instance, the genesis and evolution of saline depressions in the Tunisian Sahel were strongly determined by its subsurface, which controlled their tectonic formation and evolution [56] during the Quaternary and is currently feeding them by huge quantities of salt through aquifers convergence toward their surfaces [38,39,56]. Being in the core of the Sahel area, the MCSH system shows signs of a past tectonic activity (Figure 2) [56,61,62,92,94,96]. Based on geophysical and tectonic studies, the tectonized surface and deep subsurface were recently discussed by Ghribi [62]. Previous works [91,97] linked the tectonic structures in the Sahel area to the compressional phase originating from the convergence of the African and Eurasian plates. As it is shown in Figure 2, there are two orientations of the extensional structures: (i) the NW-SE major orientation (alignment) of Ktitir, Ouled Chamekh, and Sidi El Hani faults extends on a larger scale along the western side of the MCSH system; and (ii) the NE-SW to E-W minor orientation of Oued Mélames, Zarmdine and El Aryem faults extends on a smaller scale along the eastern side.

**Figure 3:**
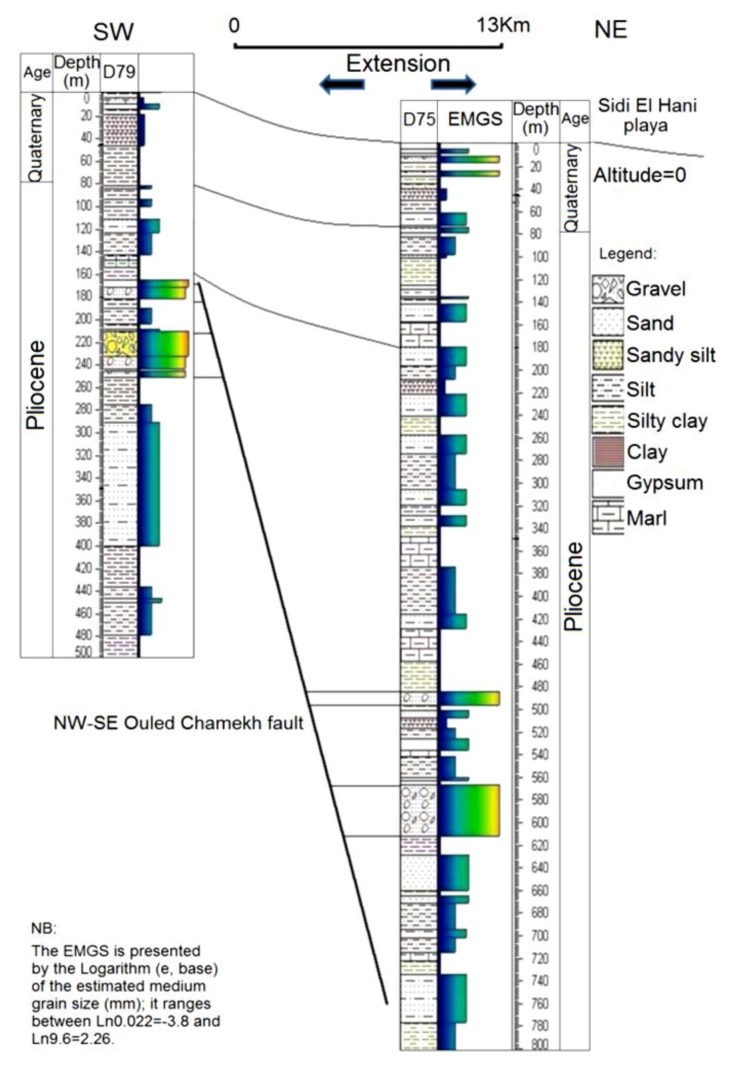
NE-SW correlation between two vibrocore drills (Figure 2; DC1) showing a syn-sedimentary fault: an extensional structure within a compressional framework.

The shallow subsurface seems also affected by these tectonic processes. Figure 3 shows a syn-sedimentary fault affecting the Pliocene series, which coincides with Segui Formation [56,61,62,92]. This formation is composed in the Sahel area of several hundred meters of clays, marls, lignites, and sandstones alternating with some (3 to 5) carbonated levels [61]. The fault of Ouled Chamekh identified in this work has an orientation between the Sidi El Hani fault and the Ktitir fault (Figure 2). This extensional structure is originated from a Neogene compressional phase. In addition, this fault may also serve as a way of water seepage to enhance spring mound formation. Thus, we find a genetic link between spring mound organization within the discharge playa and faults’ orientations. It is worth stressing that the MCSH system recorded extensional as well as compressional tectonic structures (Figure 2). This coexistence has been explained by a succession of two tectonic phases in the Sahel area [56]: a Post-Villafranchian NW-SE compression followed by a Tyrrhenian NE-SW extension controlled the opening and evolution of playas in the region. However, recent studies [91,97] suggested that comrpessional and extensional structures were coeval and originated from the very same compressional phase, which originated from the Africa-Eurasia convergence. Thus, the system experienced a coeval formation of compressional and extensional structures (Figure 2) [56,61,92,94,96,98,99,100,101,102,103] giving birth to a folded and faulted surface and subsurface (Figure 2).

**Figure 4:**
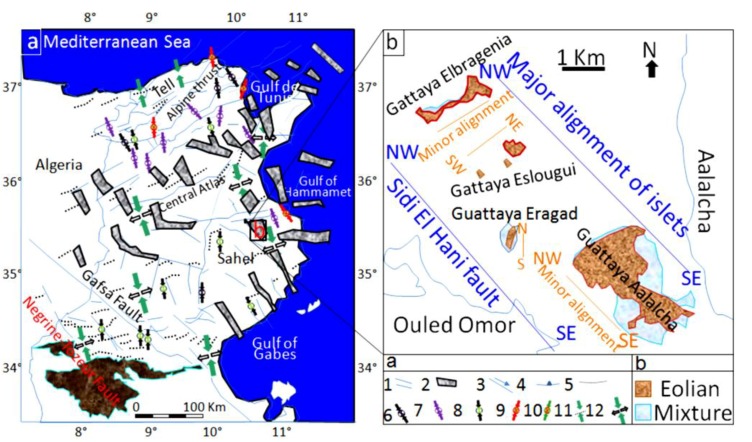
Relation between islets alignments within the Sidi El Hani discharge playa and the tectonic network in Tunisia. (**a**) Recent and current tectonic and seismotectonic map [60], modified; Zouaghi *et al.* [61]): (1) principal faults with Plio-Quaternary rejuvenation or presenting seismic activity indices; (2) graben with Plio-Quaternary rejuvenation; (3) strike-slip fault; (4) overthrust; (5) Quaternary fold or Quaternary rejuvenation; (6) direction of the P axis of on seism focal mechanism; (7) direction of the P axis of composite focal mechanism; (8) direction of compression based on the surface deformations of recent seisms; (9) direction of compression based on the historical tectonic deformations; (10) direction of the maximum horizontal constraint; (11) direction of surface principal stresses with indication of their positive (σ1) and negative (σ3) values; (**b**) Major and minor alignments of islets.

As for the recent and current tectonic activity, the system is still in the compressional phase of N–S convergence between Africa and Europe plates (Figure 4a). This convergence results in an active seismicity in the Sahel area [61,96] and in the formation of faults within depressions of Chrita and Sidi El Hani, enhancing the development of spring mounds (e.g., [35]). Figure 4b shows that the major alignment of islets in the Sidi El Hani discharge playa is compatible with alignment of recent tectonic structures recorded not only within the system but also within the Mediterranean Sea.

The study of spring mounds should take into account the tectonized zones. A tectonic influence may enhance the seepage of groundwater toward the surface of the system. This is important especially within the Chrita saline lake, where groundwater upwelling alone is not strong enough to create the springs, and therefore an exclusively hydraulic model of formation for this saline lake is unlikely.

### 4.2. Groundwater Contribution to the MCSH System

**Figure 5:**
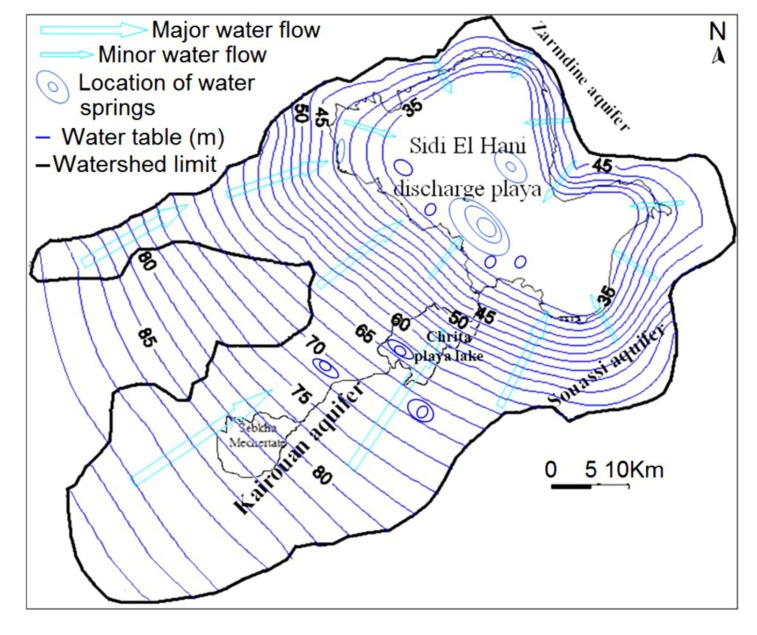
Hydrogeological map and groundwater contribution of the Mechertate-Chrita-Sidi El Hani system: water table and water flows dynamics in 2008.

The hydrogeological map of the MCSH system elaborated in 2008 (Figure 5) shows that the surrounding aquifers converge toward the Sidi El Hani discharge playa. The saline lakes of Chrita and Mechertate represent bypass zones, through which water converges to reach the basal part of the system. The map also shows that the convergence of the surrounding aquifers is more accentuated at the level of the northeastern side of the system, hence originating more springs of water within the Chrita saline lakes and the Sidi El Hani discharge playa. Recent studies [104,105] suggested an increase of deep groundwater reserves due the installation of dams in the Tunisian center, which increased the reserves of the Kairouan aquifer. Such increase accentuated water convergence and the overall salinization of the system. Aquifers within the Chrita and Sidi El Hani depressions are met at approximately 50 cm below the surface, and this near-surface water table proves vital in preserving the playa surface from wind deflation (Stokes surfaces) [106,107] and the deposition of eolian sediments, since the humidity of the surface of the playa inhibits the formation of dust by wind erosion (induration process). Thus, the permanent layer of water covering the surface of the depressions, even during the dry seasons and especially those of Chrita and Sidi El Hani, is maintained by the convergence of aquifers towards the surface, feeding the depressions with salty water.

### 4.3. Definitions and Categories of Springs and Spring Mounds at the MCSH System

After Fetter [108] and Essefi *et al.* [35] proposed a classification of springs at the MCSH system according to (1) their mode of genesis; (2) their geochemical content; (3) their activity; and (4) their stage of evolution. Mud volcanoes could have mound morphologies similar to spring mounds but they differ in the formation mechanisms. A mud volcano, also known as “hervidero” or “macaluba”, is a conical accumulation of variable admixtures of sediment resulting from eruption of wet mud and impelled upward by fluid or gas pressure. After its formation, a mud volcano may disappear or grow due to exogenous erosional or depositional factors, respectively. Conventionally, mud volcanism is linked to gas influx, especially the wrap of methane (but also of other gasses). Clear evidence of mud volcanism with gas emission is not observed at the MCSH system.

#### 4.3.1. Mode of Genesis

**Figure 6:**
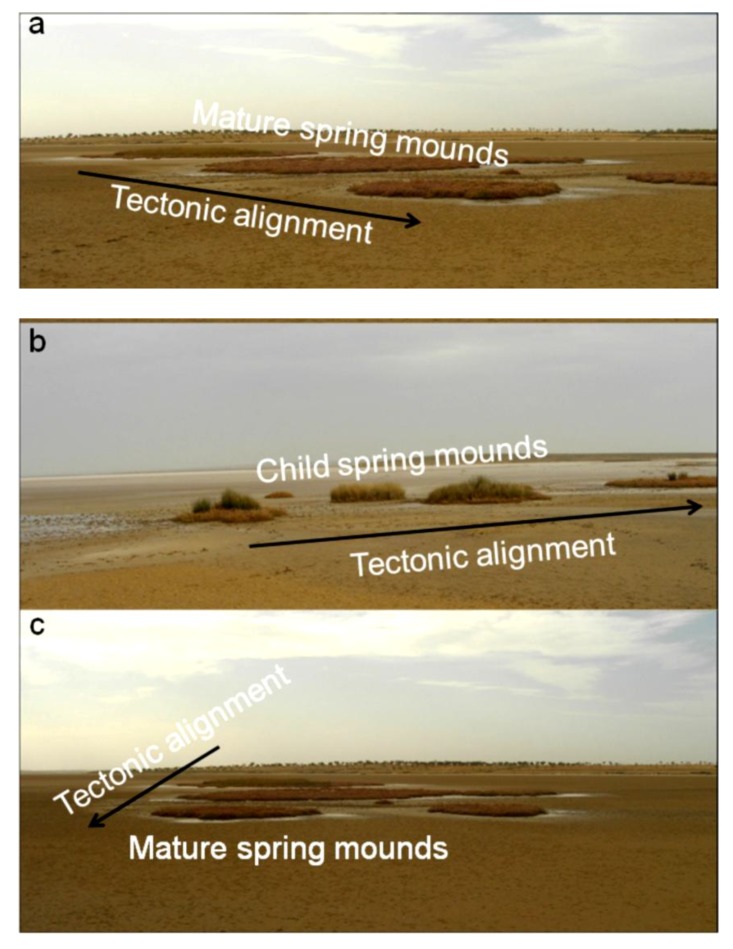
Tectonic alignment of child (**b**) and mature (**a**,**c**) spring mounds.

**Figure 7:**
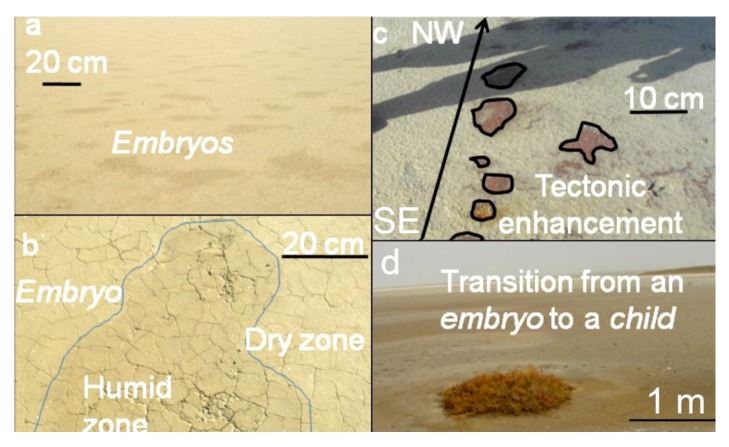
(**a**,**b**) Embryonic stage of spring mounds; (**c**) Tectonic alignment of embryos along fractures; (**d**) Transition from embryonic to child stage.

(1) Artesian springs are springs in which subsurface water ascends to the surface by means of internal pressure, generally through some fissure or other opening in the confining bed overlying the aquifer. At the core of the Sidi El Hani depression, hydraulic pressure produces emanation of water after the elimination of an impermeable clayey layer [38]. Accordingly, springs at this depression may be considered as artesian springs (Figure 6a).
(2) Gravity springs are formed under the influence of gravity, rather than internal pressure. The Kairouan aquifer is generated from the “highlands” of the so-called N-S axis draining towards the “lowlands” of Chrita and Sidi El Hani, and therefore springs mounds generated from this aquifer may be considered as gravity springs.
(3) Depression springs flow towards the surface because the surface slopes down to the water table. As the water table is located approximately at the surfaces of Chrita (this work) and Sidi El Hani [38] depressions, spring mounds at these depressions may be considered as depression springs.
(4) Perched springs arise from a body of perched water. The hydrological model previously discussed by [38] relating the Kairouan and Sidi El Hani discharge playas (Figure 19) shows that springs at Chrita and Sidi El Hani are perched.
(5) Fault springs (also known as fault-dam springs) are the result of free-flowing groundwater onto the land surface surging from a previously faulted area that brought a permeable bed into contact with a less-permeable layer. Due to the major role of faulting in enhancing formation and development of spring mounds at the Chrita (this work) and Sidi El Hani [35] depressions, these spring mounds may be considered as fault spring mounds (Figure 6).
(6) Contact springs are formed due to the gravity flow of water from a groundwater source to the land surface, from permeable strata overlying impermeable strata that prevent or delay percolation. Spring mounds at the Chrita playa surface allow water seepage through springs and planar surfaces. They may be considered, hence, as contact springs.
(7) Fracture springs are the result of the natural flow of groundwater surging from joints or other fractures in bedrock, and may be flowing at several different locations along the fracture. Due to the tectonic activity, fracture springs are obvious manifestation on both the Chrita and Sidi El Hani depressions (e.g., [35]) (Figure 7c).

#### 4.3.2. Geochemical Content

(1) Brine springs are salt-water springs. The geochemical analysis of the discharging water from a spring at Sidi El Hani (Figure 11c; W2) shows a salinity of 7.25 g/L. This spring may be considered as a brine spring.
(2) Mineral (gypsum, chloride, magnesium) springs are springs whose water has a definite taste due to the high concentration of a specific mineral. The spring may be named according to the geochemistry of its water. At spring mounds of the Sidi El Hani discharge playa, the weight of chloride represents more than 50% of the total ions. Hence, they are chloride springs.
(3) Carbonate spring is a spring containing dissolved carbon dioxide gas.
(4) Sulfur spring is a spring containing sulfur compounds such as hydrogen sulfide content.
(5) Mud pot (also known as painted pot, sulfur-mud pool) is a type of hot spring, which contains boiling mud, typically sulfurous and often multicolored. They tend to be associated with geysers and other hot springs in volcanic zones. The latest three types were not identified at depressions of the MCSH system.

#### 4.3.3. Activity

(1) Perennial springs flow continuously, because of a hydrogeologic convergence of deep aquifers. At the MCSH system, deep aquifers convergence guarantees the activity of some springs [38].
(2) Intermittent springs cease flowing after a long dry spell and flow again after heavy rains.
(3) Periodic springs flow periodically, apparently due to natural siphon action. Though mentioned in the literature (e.g., [108]), the two last types were not identified in our system.
(4) Seepage springs (also known as weeping springs) are characterized for showing small discharges. The activity of this type is influenced by the hydraulic pressure, the tectonic activity, and the lithology of the playa surface. The vegetation or chemical precipitates can provide clues as to the presence of springs and seeps. Vegetation includes salt-tolerant phreatophytes (e.g., Figure 11) such as willow, cottonwood, mesquite, salt grass, and greasewood. At the mouth of the springs, travertine limestone concretionary deposits may be formed (e.g., sebkhas of Chrita and Sidi El Hani). Highly saline groundwater springs (brine, mineral or carbonate springs) can result in the formation of saline soils, playas, salinas, and salt precipitates (e.g., sebkhas of Sidi El Hani and Chrita).

#### 4.3.4. Stage of Evolution

The size of a spring mound varies from few meters to few kilometers. These different sizes indicate different evolutionary stages. Essefi *et al.* [35] argued that the development of spring mounds playa is a slow and continuous process following successive stages, rather than a rapid construction. Spring mounds may be found in different stages, which were named, in chronological order, *abortive*, *embryonic*, *child*, *mature* (adult), and *islet* (old) stages.

(1) At the *abortive* stage (Figure 6a), thousands of abortive spring mounds chaotically form. The majority of these spring mounds are aborted due to a weak hydraulic pressure and/or the absence of tectonic enhancement. The size of this type may be considered less than one meter.
(2) At the *embryonic* stage (Figure 6b,c), the tectonic activity and hydraulic pressure result in the formation of spring mounds along a preferential orientation. The size of this type may be considered between 1 m and 2 m.

**Figure 8:**
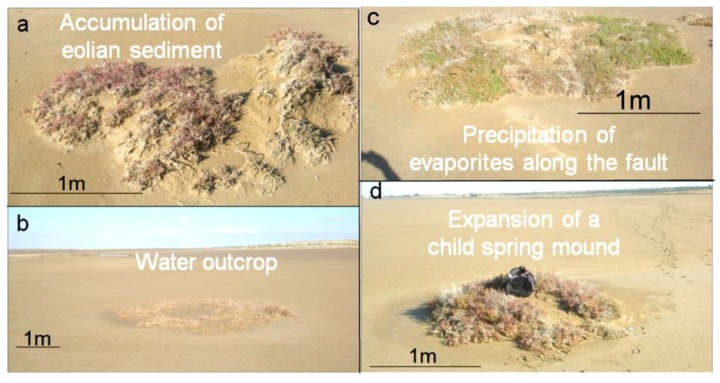
(**a**) Accumulation of eolian sediment on a *child* spring mound; (**b**) Active *child* artesian spring mounds; (**c**) Accumulation of travertine on a fault *child* spring mound; (**d**) Continuous seep along a *child* spring mound.

At the child stage (Figure 8), eolian sedimentation and geochemical precipitation compete. Consequently, simultaneous deposition of evaporites and eolian sediment is observed. The size of this type may be considered between 2 m and 10 m.

(3) At the *mature* stage (Figure 11), the spring mound is covered with eolian sediments, acting as an obstacle and collecting more eolian sediments. The size of this type may be considered between 10 m and 80 m.
(4) At the *islet* stage, eolian deposition dominates the system, and the salty soil is completely buried. The size of this type may be considered more than 80 m.

The occurrence of these types of spring mounds was identified at Sidi El Hani discharge playa [35]. In this paper, the terminology combines the mode of genesis (e.g., artesian or fault) and the stage of evolution (e.g., child or mature).

### 4.4. Spring Mounds Detected by Satellite Images

**Figure 9:**
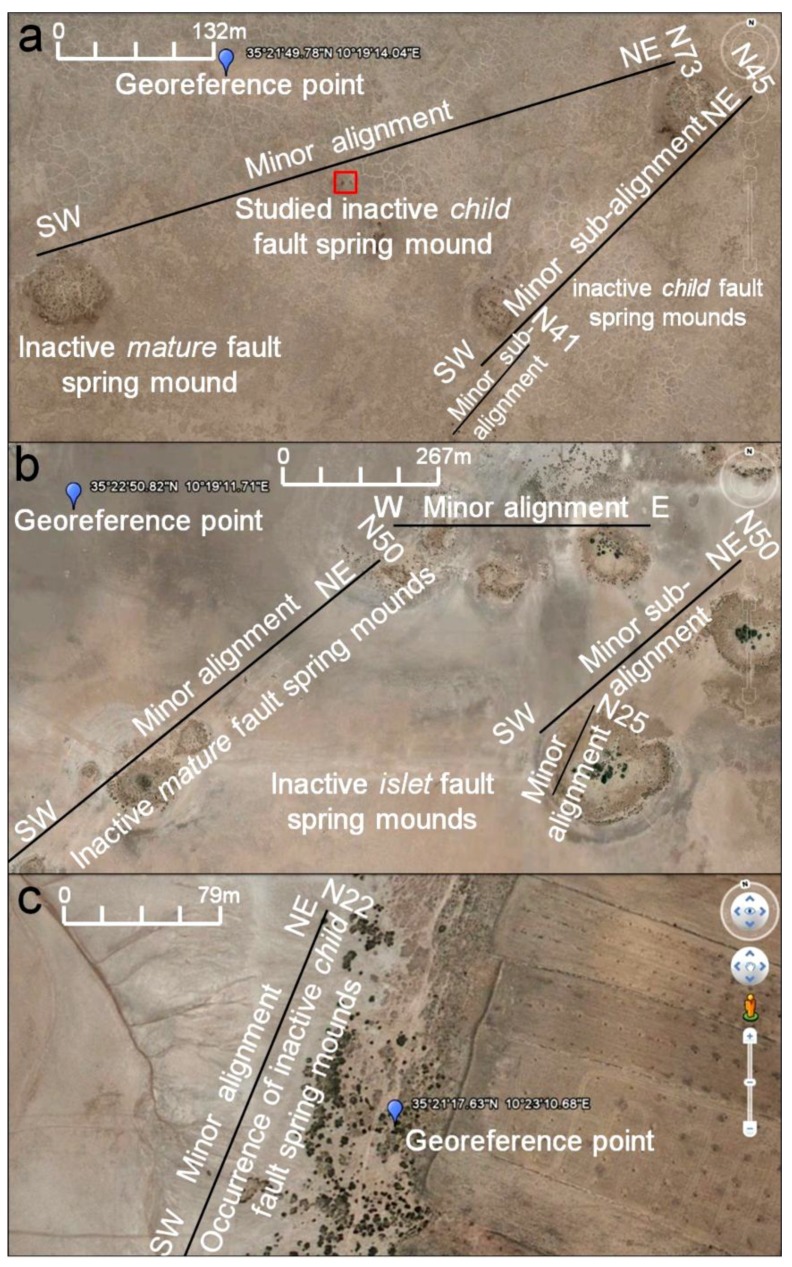
*Child*, *mature*, and *islet* fault spring mounds in the Chrita saline lake oriented according to the minor tectonic alignment of the Sahel area. Google Earth images, major axis (**a**) 132 m; (**b**) 267 m; (**c**) 79 m.

Within the Chrita saline lake, *mature*, *child*, and *islet* spring mounds were identified at different scales (132 m, 267 m, 79 m; Figure 9). They seem to be organized according to NE-SW to N-S lines. This alignment is compatible with the NE-SW to N-S minor alignment of the tectonic map of Tunisia (Figure 4a). As it is shown in Figure 9, the alignment varies along Chrita depression between N73, N50, and N22. Further, the principal tectonic alignment may be divided into 2 or 3 sub-alignments. Such organization proves that these structures are genetically linked, and supports surface-subsurface connectivity. Water seepage and/or upwelling should have taken place through a NE-SW to N-S subsurface faults. Thus, these fault spring mounds identified on the surface of Chrita saline lake are originated from past and present tectonic activities. Past activities, which were mainly due to the convergence of African and Eurasian plates, were responsible for the folding of the Sahel and the opening of these saline environments in eastern Tunisia [38,56,97]. Current tectonic activity originated from Europe and Africa convergence causes an active seismicity [61,96] and an obvious tectonic faulting noticed on depressions’ surfaces (e.g., [35]). Hydrogeologically, since Chrita saline lake represents a bypass zone of water flows (Figure 5), the hydraulic pressure is far from being strong to impose the occurrence of artesian or at least artesian-fault spring mounds. Instead, inactive faults spring mounds occur on the surface of Chrita saline lake. This inactivity is expressed by the absence of an intense vegetative cover. It is worth mentioning that spring mound activity may be inferred from satellite images. On active spring mounds, vegetation flourishes and gives a dark coloration to satellite images; while inactive spring mounds are covered by eolian sediment giving a clear tinge to satellite images. We notice also that a minor alignment is set along an inactive *islet* fault spring mounds (Figure 9b). This *islet* may be the result of a fusion of small spring mounds, which might have started as isolated fault spring mounds; then, their evolution through eolian accumulation resulted in their merging in one *islet*.

**Figure 10:**
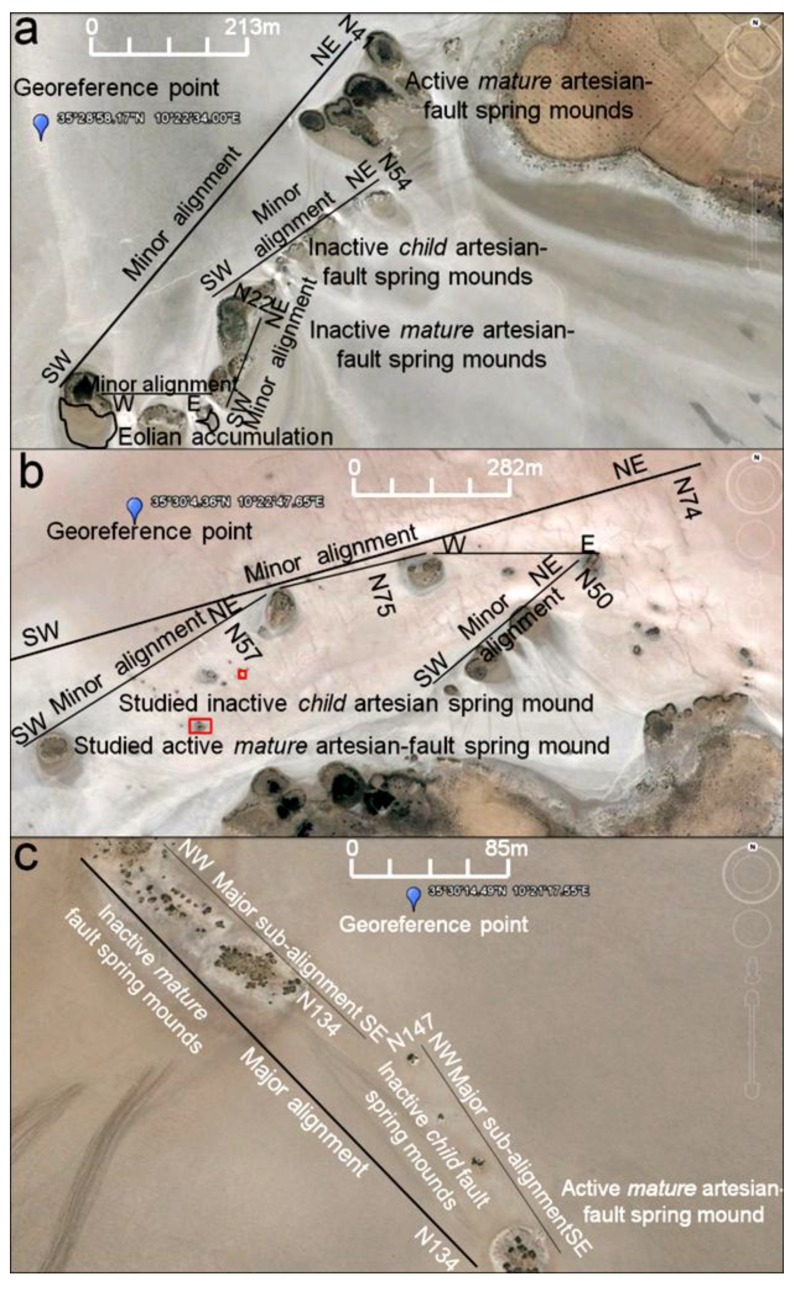
Spring mounds in the Sidi El Hani discharge playa. Google Earth images, major axis 213 m (**a**); 282 m (**b**); 85 m (**c**).

Within the Sidi El Hani discharge playa, active artesian-fault and inactive fault (*mature* and *child*) spring mounds were also identified at scales of 213 m, 282 m, and 85 m (Figure 10). These spring mounds seem organized along the NE-SW to E-W minor alignment (Figure 10a,b) and according to the NW-SE major orientation (Figure 10c) of the tectonic map of Tunisia (Figure 4a). Figure 10a shows that the sub-alignment of active *mature* artesian-fault spring mounds is parallel to the minor alignment N41. While the sub-alignment of the inactive *child* fault spring mounds is N54. The inactive *mature* fault spring mounds are oriented N22. Some E-W active and inactive *mature* fault spring mounds accumulate eolian sedimentation around them. Figure 10b shows that inactive *child* fault and active *mature* artesian-fault spring mounds follow a minor alignment of N74, which is approximately equal to the mean of sub-alignments N75, N57, N50 and N90 (E-W). The major alignment of inactive *child* and *mature* fault spring mounds is N134 (Figure 10c).

On the other hand, other *child* artesian spring mounds (currently inactive) seem to have a chaotic distribution (Figure 10b). Such organization proves that these structures have been originated from a weak hydraulic pressure, which was not enough to impose water emanation. Furthermore, the size of these spring mounds would not increase and they may be blocked at an initial stage of development of a spring mound [35].

The tectonic activity identified on a macro-scale by satellite images has been also detected during our field work along depression surfaces (see Section 4.5). The tectonic activity enhances spring mound formation by increasing the permeability of the sebkha material. The occurrence of spring mounds in both Chrita and Sidi El Hani depressions is also promoted by the low viscosity of the material, which is composed of 40% water.

### 4.5. Spring Mounds Identified during Field Campaigns

The results of our field reconnaissance show that the fault spring mounds at the Chrita saline lake are inactive because the hydrogeologic pressure is low. However, in spite of the absence of an obvious activity of spring mounds, Oued Chrita (local name meaning “the belt”), which is connecting the Chrita saline lake and the Sidi El Hani discharge playa, drains permanently even in absence of any rain. This permanent activity of Oued Chrita is strong evidence of water coming up from subsurface. The emanation is materialized by a slow seepage along the total surface of the saline lake rather than a localized upwelling through spring mounds. Seepage is enhanced by the permeable sandy sediment.

**Figure 11:**
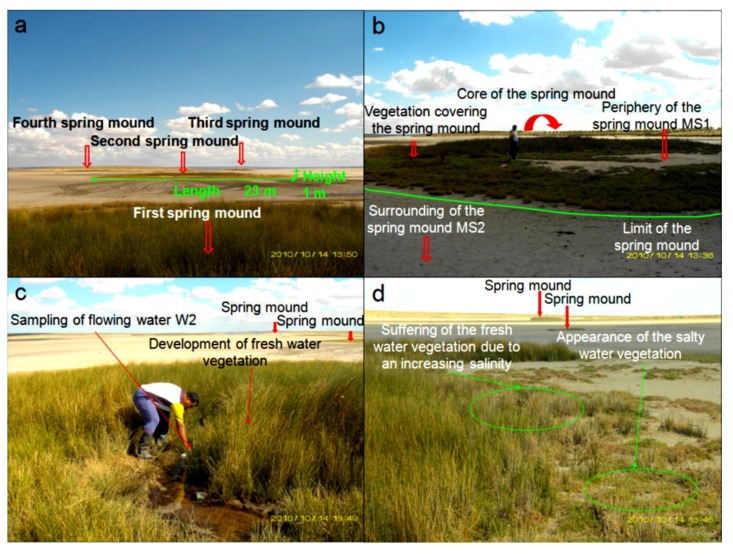
Alignment (**a**) and morphology (**b**), of spring mounds located in the Sidi El Hani discharge playa; Sampling (**c**) and variability of the vegetation with an increasing salinization on an active spring mound from an active spring mound (**d**).

In the western side of the Sidi El Hani discharge playa, we identified some spring mounds (Figure 11). They have different lengths ranging from 3 m to 40 m, while their heights barely reach one meter. Sometimes, these spring mounds are occupied by central springs including water surges (Figure 11b,c). These active spring mounds dissipate the water through direct emanation from their centers and through seepage from their peripheries. Sometimes, the pressure of the water is not enough to generate emanation through a central spring. Hence, these inactive spring mounds dissipate the water through seepage from their peripheries. Accordingly, the nearby salty water creates a layer of precipitated travertine. Contrary to spring mound activity at Chrita, spring mound activity at the Sidi El Hani discharge playa is enhanced by the tectonic activity and hydraulic pressure. When a spring mound is enhanced by a fault, it may develop towards a mature and active spring mound [35]. Otherwise, it remains at younger, less-developed stages [35]. Thus, we can hypothesize that inactive spring mounds are artesian spring mounds originated by a weak hydraulic pressure. On the other hand, active artesian spring mounds enhanced by tectonic activity may be defined as artesian-fault spring mounds.

As for the vegetation cover of the spring mounds (Figure 11), plants requiring relatively fresh water occupy the peripheries (Figure 11c,d). Closer to the discharge playa (Figure 11d), fresh water-requiring vegetation is substituted for vegetation that tolerates more salinity.

### 4.6. Models of Spring Mound Activity and Evolution

The stage of evolution (*embryos*, *child*, *mature* or *islet*), activity (active or inactive) and maturation (eolian accumulation and geochemical precipitation) of spring mounds are the result of their tectonic, hydrogeologic, and sedimentary contexts. The tectonic and hydraulic factors enhance their primary formation. Then, the eolian sedimentation leads their evolution toward distinctive islets.

#### 4.6.1. Inactive *Child* Spring Mound (Seep) in the Chrita Saline Lake

**Figure 12:**
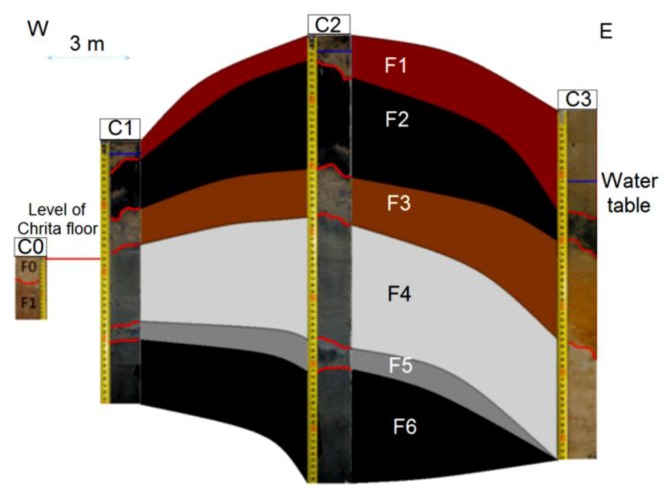
Facies identification and correlation between cores of an inactive *child* spring mound located in the Chrita saline lake.

This *child* spring mound is located in the Chrita saline lake. Though it is inactive (only seepage), it may be noticed by the naked eye. Its height is approximately 20 cm and its length reaches 18 m (Figure 14). Three drill cores were taken along an E-W profile. Based on the Visual Core Description (VCD), six different facies (Fx) were identified along the three cores (Figure 12). F1 is characterized by its red color, and is an eolian facies deposited after the formation of the *child* spring mound. The sediment comes from the system itself or from its vicinities [53]. F2 is characterized by its black color, which reflects a high content of organic matter and/or high degree of confinement. The organic matter may be autochthonous due bacterial activity and/or migrating fluid [109]. Hence, this facies is probably of endogenous origin or it is deposited under calm hydrodynamics allowing the conservation of the organic matter. F1 protects this organic matter from hard conditions that would impede bacterial activity or destroy the conserved organic matter. F3 is similar to F1 with a slight difference in terms of redness, which reflects its richness with iron oxide. F4 is grey in the western and middle cores (C1, C2), while it is beige at the eastern core (C3). The differences are likely due to the eolian accumulation at the northern side of the system. F5 is darker than F4, reflecting a dominance of the endogenous factor at the expense of exogenous factor in the southern core. F5 is absent in the southern core (C3), where the eolian factor overcomes the endogenous factors. F6 is also detected only in the northern and middle cores, and is darker than the previous facies, indicating a decrease in eolian sedimentation. Our data suggest that the formation of the *child* spring mound located in the Chrita saline lake is controlled by the interplay of endogenous and exogenous factors. Sometimes one factor dominates over the other, and sometimes the two factors compete to produce a kind of mixture. The correlation between different facies allows a 2D modeling of this spring mound (Figure 12).

**Figure 13:**
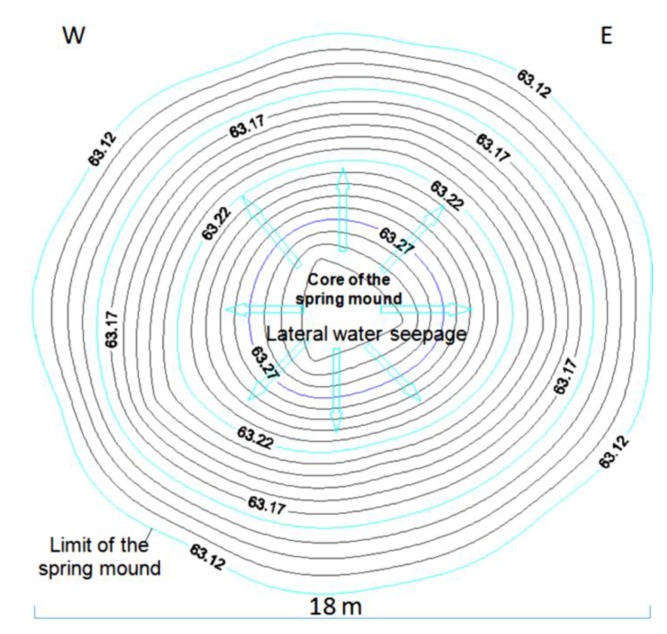
Micro-hydrogeological map (water seepage) of an inactive *child* spring mound located in the Chrita saline lake.

The hydrogeological micro-map (Figure 13) shows that the water table varies between 63.3 m and 63.1 m. Such gradient (0.2 m) allows mapping the water flows from the center to the periphery of the *child* spring mound. Nevertheless, these flows are not strong enough to force discharging of water at the level of the center of the spring mound. Instead, water saps toward the floor of the saline lake from the peripheries of the *child* spring mound.

**Figure 14:**
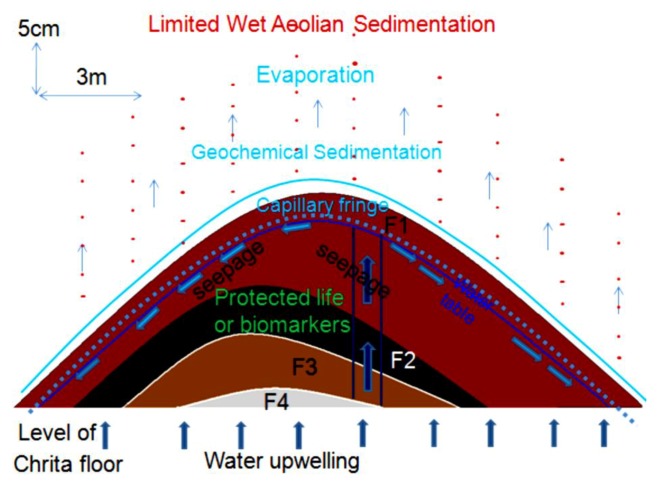
Conceptual model of an inactive *child* spring mound located in the Chrita saline lake: simplified activity model.

The facies identification and the micro-hydrogeological studies allow the convolution of a hydrodynamic model showing water circulation and facies extension (Figure 14): on one hand, water circulates from the bottom upward by upwelling and then, when it faces a strong obstacle, it laterally saps toward the periphery of the *child* spring mound; on the other hand, sedimentary facies stretch along the *child* spring mound with curved shapes.

**Figure 15:**
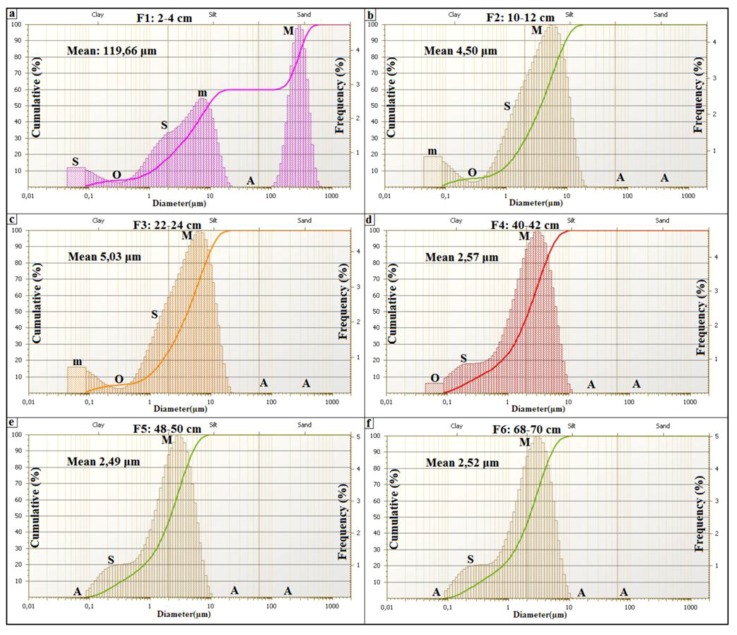
Grain size distribution along a drill core from a spring mound located in the Chrita saline lake.

The grain size distribution (Figure 15) of the drill core in the middle of the *child* spring mound (Figure 12; drill core C2) shows the interplay of eolian, hydraulic, and geochemical components. The grain size distribution of F1 shows a dominance of the coarse (Mean: 199.66 µm) eolian sedimentation (Figure 15a). F1 is characterized by a primary grain size (M: *ca.* 315 µm) as indication of the coarse eolian sedimentation. F1 also show a secondary grain size (m: *ca.* 7 µm), a shoulder (S: *ca*. 1.5 µm), an occluded (O: *ca.* 0.1–1 µm) as indication of an occlusion of hydraulic sedimentation, and an absent (A: *ca.* 20–100 µm). The geochemical fraction lower than 0.1 µm behaves as second shoulder. In this facies, the eolian sedimentation takes the dominance. The second (Figure 15b) and third (Figure 15c) facies show a finer grain size (Mean ≈ 5 µm). F2 and F3 are characterized by primary modes (M: *ca.* 7 µm) as indication of fine eolian sedimentation, an occluded (O: *ca*. 0.1–1 µm) as indication of occlusion of hydraulic sedimentation, and an absent (A: *ca*. 20–1000 µm) as indication of absence of coarse eolian sedimentation; the geochemical fraction lower than 0.1 µm behaves as secondary mode. In these facies, the geochemical sedimentation overcomes the coarse eolian sedimentation and balances the fine eolian one. The grain size fining continues with F4 (Figure 15d), F5 (Figure 15e) and F6 (Figure 15f) (Mean ≈ 2.5 µm). Comparably with the previous facies, they are characterized by the occlusion (F4) and the absence (F5 and F6) of the geochemical precipitation. We note also the appearance of the fine hydraulic sedimentation as shoulder (S: *ca.* 0.1–1 µm) in the three facies. These facies belong to the filling of the Chrita saline lake, where the deposition is controlled by the interplay between the eolian and the hydraulic sedimentation.

#### 4.6.2. Inactive *child* Spring Mound in the Sidi El Hani Discharge Playa

**Figure 16:**
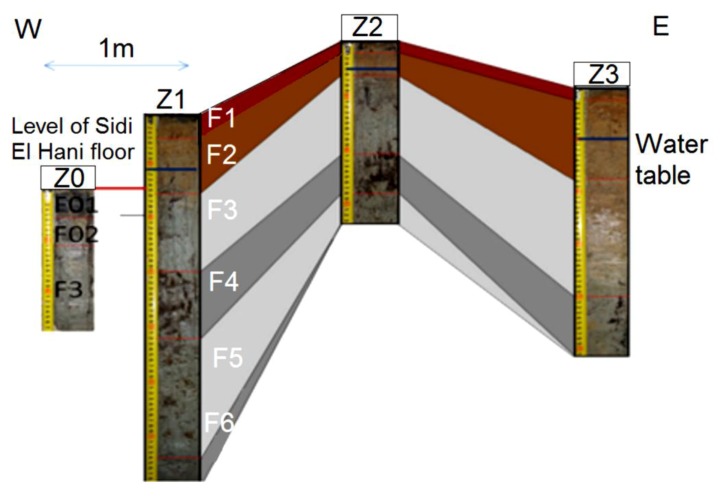
Inactive spring mound located in the Sidi El Hani discharge playa: facies identification and correlation between cores.

This *child* spring mound is located in the western side of the Sidi El Hani discharge playa, and can be noticed by the naked eye and on satellite images. Its height is approximately 25 cm and its length reaches 6 m. The extraction and examination of three cores allows the identification of six different facies (Figure 16), but they do not all appear in the three cores. Due to their eolian origin, F1 and F2 are characterized by their red color. F3 and F4 are beige, showing hence a tendency toward their origin as autochthonous sediments. F5 and F6 represent the filling of the discharge in itself. Thus, the formation of this *child* spring mound involves the interplay of the two factors: autochthonous sediments and eolian deposition.

**Figure 17:**
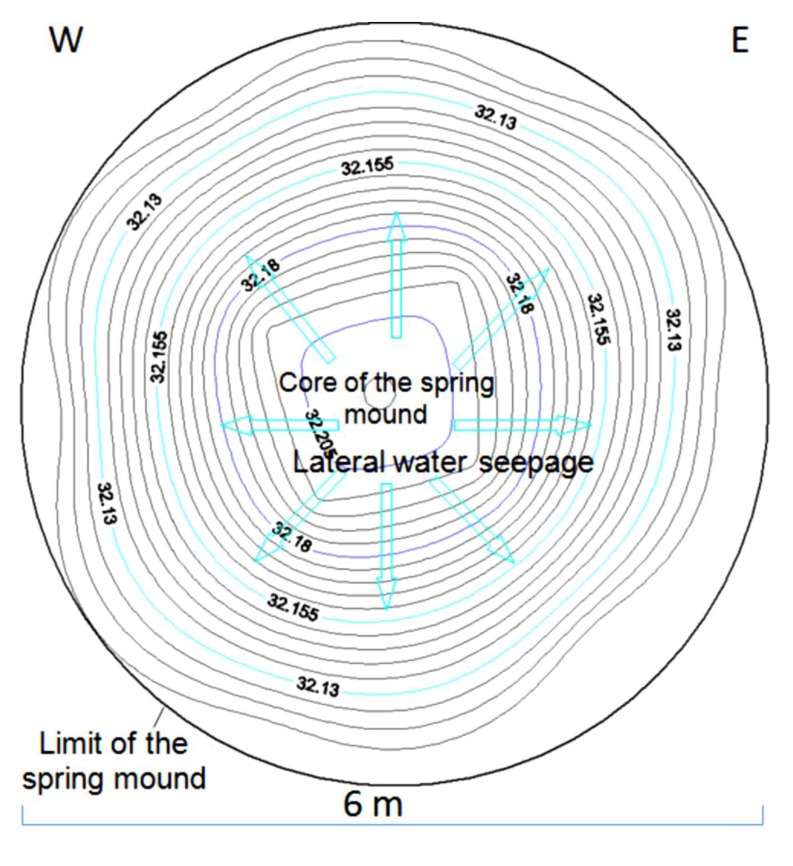
Inactive spring mound located in the Sidi El Hani discharge playa: hydrogeological map (water seepage).

The detailed hydrogeological map (Figure 17) shows that the water table in the mound varies between 32.11 m and 32.21 m. Such gradient (0.1 m) allows the displacement of water flows from the center to the periphery of the spring mound. However, these flows are not strong enough to force the emanation of water at the center of the spring mound.

**Figure 18:**
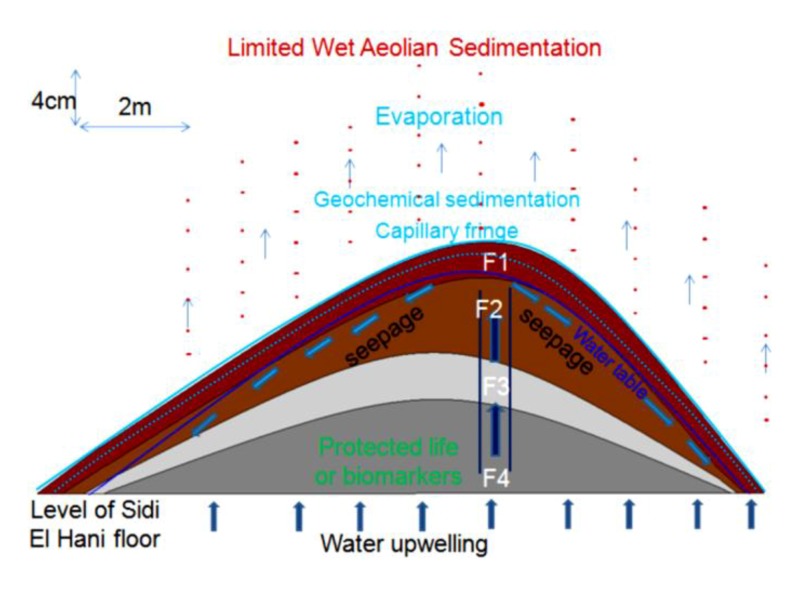
Conceptual model of an inactive *child* spring mound located in the Sidi El Hani discharge playa: simplified activity model.

The facies identification, the sedimentary analyses, and the micro-hydrogeological studies allow the convolution of a hydrodynamic model showing water circulation and facies extension (Figure 18). Water moves from the bottom upwards by upwelling circulation. Then, when it faces a strong obstacle, it laterally saps toward the periphery of the spring mound. The center of the spring mounds shows the precipitation of powdery gypsum because of the increase of the water table and capillary fringe. Comparably to the active spring mound in the Sidi El Hani discharge playa, which is located by no more than 100 m distance, this inactive spring mound has the same hydrogeologic context, but the gradient within it is smaller than the active spring mound. This difference is probably due to a local tectonic condition, in which a fault under the active spring mound may increase its permeability and its hydrogeologic dynamics. The active spring mound is probably located on the active component of the Sidi El Hani deep fault [62], which may reach shallower levels (as it is shown in the tectonics study; see Figure 3). The dynamic evolution is controlled by sedimentary processes, because the water table, which enhances the evolution of the large (23 m) active spring mound toward an islet, is absent in the case of the small (6 m) inactive spring mound. Thus, islets in the Sidi El Hani discharge playa may be interpreted as the result of surface-breaking thrust and reverse faults. The faulting depths assumed from the lobate scarps of the MCSH system are between a few kilometers (e.g., [62]) to a few hundreds of meters (this study; Figure 3).

**Figure 19:**
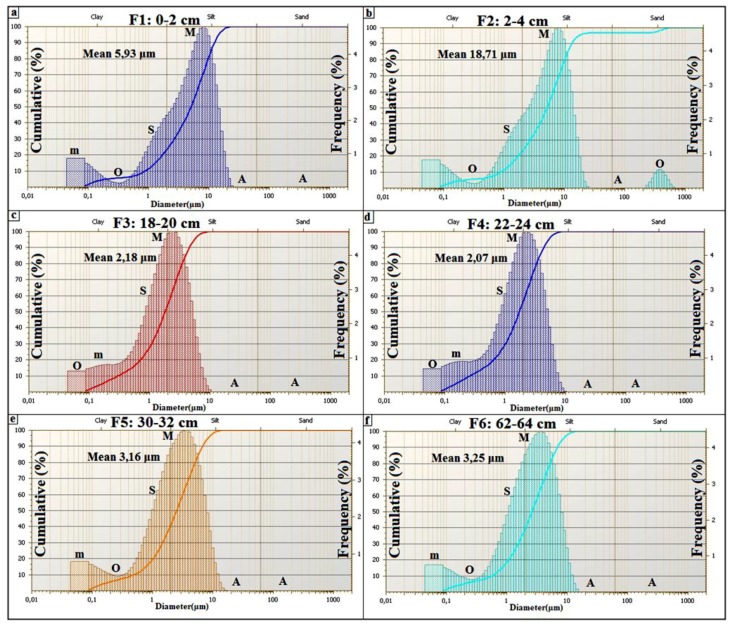
Grain size distribution along a drill core from an inactive spring mound located in the Sidi El Hani discharge playa.

The grain size distribution of core 2 from the inactive spring mound at Sidi El Hani (Figure 19) shows the interplay of eolian, hydraulic, and geochemical components. The grain size distribution of the first facies (Figure 19a) shows the dominance of the fine (Mean: 5.93 µm) eolian sedimentation. F1 is characterized by a primary mode (M: *ca.* 10 µm) as indication of the fine eolian sedimentation, a secondary mode (m: *ca.* 0.1 µm) as indication of geochemical sedimentation, a shoulder (S: *ca.* 1.5 µm), an occluded (O: *ca.* 0.1–1 µm) as indication of the occlusion of hydraulic sedimentation, and an absent (A: *ca.* 25–250 µm). Comparably to F1, the grain size distribution of F2 (Figure 19b) shows the noticeable coarsening (Mean: 18.71 µm). However occluded, the coarse eolian sedimentation is present (O: *ca.* 315 µm). F3 and F4 (Figure 19c,d) show a grain size fining (Mean ≈ 2 µm). They are characterized by primary modes (M: *ca.* 2 µm) as indication of fine hydraulic sedimentation. The secondary mode (m: *ca.* 0.1–1 µm) indicates an appearance of the hydraulic sedimentation, while an absent (A: *ca.* 20–1000 µm) indicates the absence of coarse eolian and hydraulic sedimentation. The geochemical fraction lower than 0.1 µm behaves as shoulder. In these facies, the geochemical sedimentation overcomes the eolian sedimentation, and competes with the hydraulic one. F5 and F6 (Figure 19e,f) show a slight grain size coarsening (Mean ≈ 3 µm). They are characterized by primary modes (M: *ca.* 5 µm) as indication of fine eolian sedimentation, an occluded (O: *ca.* 0.1–1 µm) as indication of occlusion of hydraulic sedimentation, and an absent (A: *ca.* 20–1000 µm) as indication of absence of coarse eolian sedimentation; the geochemical fraction lower than 0.1 µm behaves as secondary mode. In these facies, the geochemical sedimentation overcomes the coarse eolian sedimentation and balances the fine eolian one.

#### 4.6.3. Active Spring Mound in the Sidi El Hani Discharge Playa

**Figure 20:**
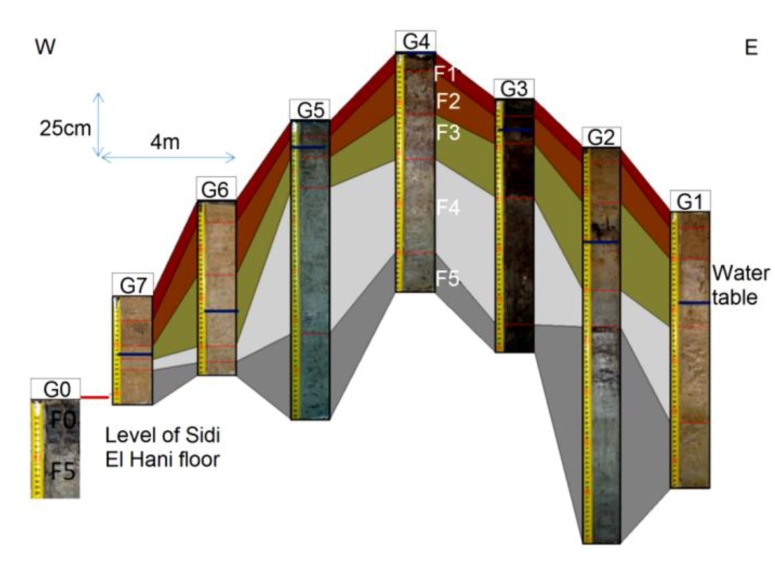
Active spring mound located in the Sidi El Hani discharge playa: facies identification and correlation between cores.

This spring mound is located in the western side of the Sidi El Hani discharge playa, located at a distance of less than 100 m from the inactive spring mound. Its height is approximately 90 cm and its length reaches 23 m (Figure 22). The elaboration of seven drill cores allows the identification of five different facies (Figure 20). F1 is characterized by its red color, probably due to its eolian origin. As it is case in the Chrita saline lake, this eolian facies was deposited after the formation of the spring mound. Though slightly different from F1, F2 seems to have the same eolian origin. Both facies hence are the result of dominance of the allochthonous sedimentation at the expense of autochthonous processes. F3 shows signs of endogenous origin, or it is deposited under calm hydrodynamics allowing the conservation of the organic matter. In F4 the endogenous factors are dominant, as can be observed in the grey sediment. F5 further increases the tendency toward the dominance of the endogenous factor at the expense of exogenous factor, showing a total absence of the eolian sedimentation.

**Figure 21:**
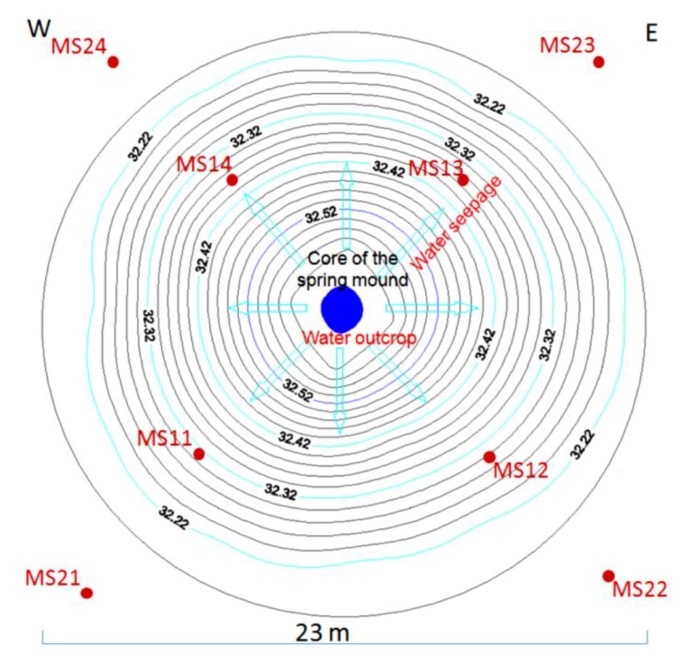
Active spring mound located in the Sidi El Hani discharge playa: hydrogeological map (water flow and seepage).

The hydrogeological micro-map (Figure 21) shows that the water table varies between 32.6 m and 32.2 m. Such gradient (0.4 m) allows the flow of water from the center to the periphery of the spring mound. Further, these flows are strong enough to force discharging of water at the level of the center of the spring mound.

**Figure 22:**
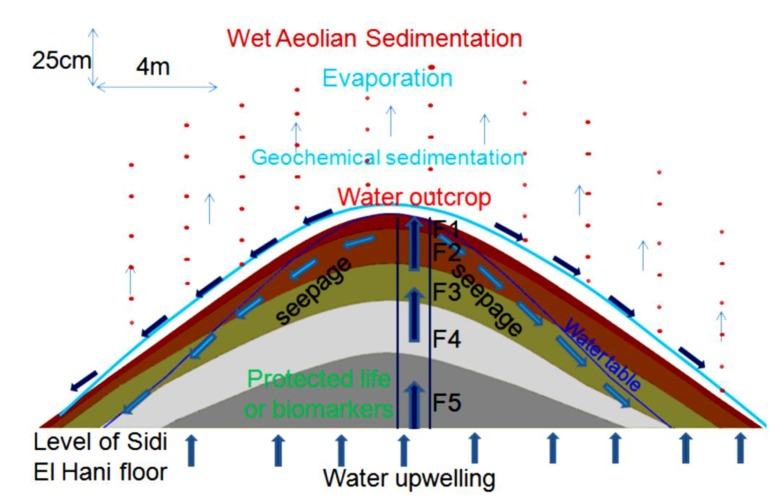
Active spring mound located in the Sidi El Hani discharge playa: simplified activity model.

The facies identification and the sedimentologic and the micro-hydrogeological studies allow the convolution of a hydrodynamic model showing water circulation and facies extension (Figure 22c). Water circulates from the bottom upward by upwelling, and finally emanates and feeds the discharge playa by saline water.

**Figure 23:**
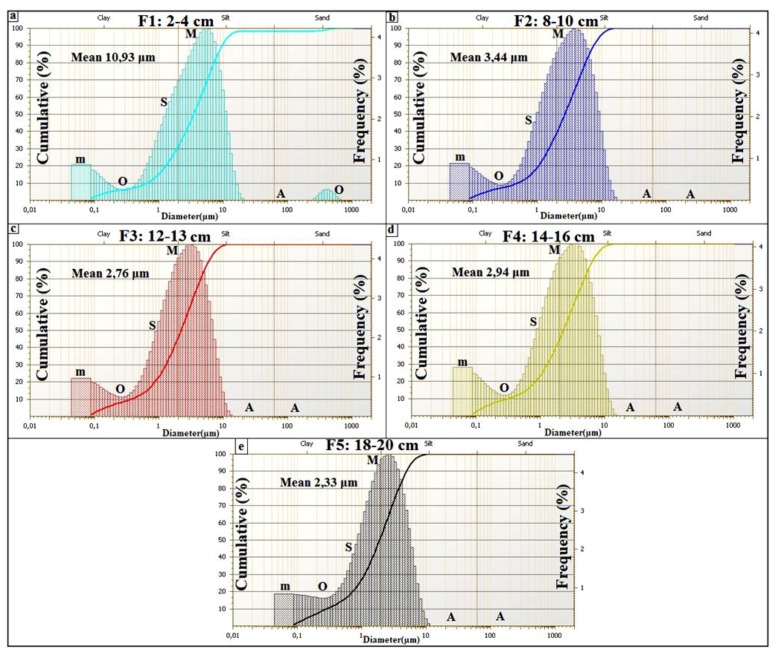
Grain size distribution along a drill core from an active spring mound located in the Sidi El Hani discharge playa.

The grain size distribution of core 7 from the active spring mound (Figure 23) shows the interplay of the eolian, the hydraulic, and the geochemical components. The grain size distribution of the first facies (Figure 23a) shows the dominance of the fine (Mean: 10.93 µm) eolian sedimentation. F1 is characterized by a primary mode (M: *ca.* 7 µm) as indication of the fine eolian sedimentation, a secondary mode (m: *ca.* 0.1 µm) as indication of geochemical sedimentation, a shoulder (S: *ca.* 1.5 µm), an occluded (O: *ca.* 0.1–1 µm) as indication of the occlusion of hydraulic sedimentation, and an absent (A: *ca.* 10–250 µm). However present, the coarse eolian sedimentation is occluded (O: *ca.* 315 µm). In this facies, the fine eolian sedimentation is dominant. The fourth remaining facies (Figure 23b–e) show finer grain sizes (Mean ≈ 2.8 µm). They are characterized by primary modes (M: *ca.* 7 µm) as indication of fine eolian sedimentation, an occluded (O: *ca.* 0.1–1 µm) as indication of occlusion of hydraulic sedimentation, and an absent (A: *ca.* 20–1000 µm) as indication of absence of coarse eolian sedimentation. The geochemical fraction lower than 0.1 µm behaves as a secondary mode. In these facies, the geochemical sedimentation overcomes the coarse eolian sedimentation and balances the fine eolian one.

##### X-ray Diffraction Data

To the naked eye, the sedimentary content of these spring mounds appears to be different from their surroundings, an observation reinforced by the different values of the magnetic susceptibility (Table 1) of the sediment of the spring mound (Figure 21, Table 1, MS11, MS12, MS13 and MS14) and its surroundings (Figure 21, Table 1, MS21, MS22, MS23 and MS24). Hence, we suggest that the surface of the spring mound having high values of magnetic susceptibility is a site of eolian sedimentation. The continuity of this sedimentation, which is itself a result of the capillary fringe and the induration process, will result in the development of this spring toward an islet.

**Figure 24:**
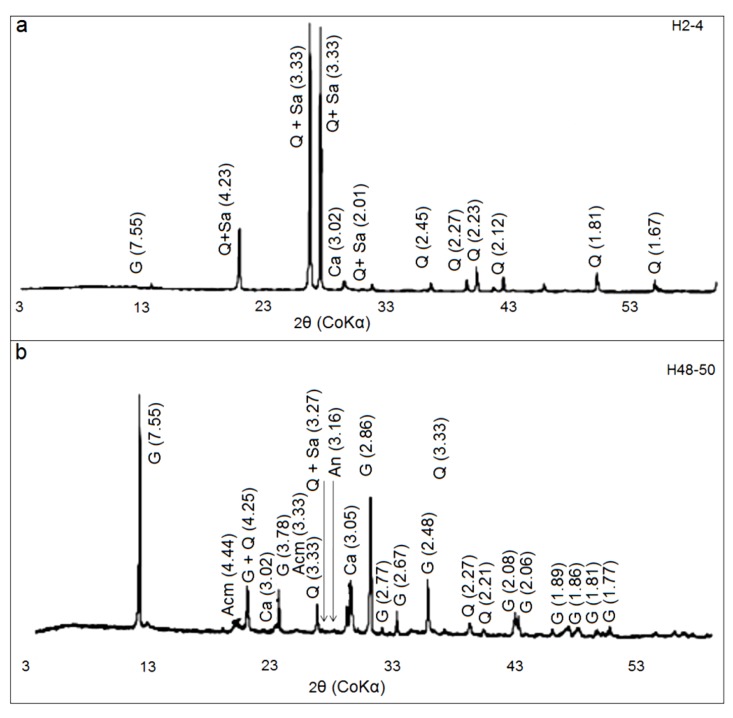
X-ray diffraction patterns (CoKα radiation) of H2-4 (**a**) and H48-50 (**b**) bulk rock samples. (G: Gypsum; Acm: All clay minerals; Q: Quartz; Or: Orthose; An: Anorthite; Sa: Sanidine; Ca: Calcite).

The XRD patterns of bulk rock of two specimens (H2-4 and H48-50) indicated that they are mainly composed of gypsum, calcite, quartz, feldspars, and all clay minerals. Their strong reflections appear at 7.55 Å, 3.03 Å, 3.33 Å, (3.18 Å, 3.24 Å, and 3.31 Å), and 4.44 Å respectively (Figure 24). The feldspar minerals are a mixture of anorthite, orthose, and sanidine their strong reflections, are observed for the H2-4 and H48-50 bulk samples (Figure 24), at 3.18 Å, 3.24 Å and 3.31 Å, respectively. For bulk rock samples, the quartz, sanidine and orthose are the main minerals of the sample H2-4, but the gypsum mineral is the major phase of the sample H48-50 associated to anorthite and sanidine minerals.

**Table 1:** Low Frequency susceptibility, High Frequency susceptibility and Frequency-dependent susceptibility of sediments from the active spring mound in the Sidi El Hani discharge playa and from its surroundings.

Sample	LF Susceptibility 10^−6^ (SI)	HF Susceptibility 10^−6^ (SI)	Frequency-Dependent Susceptibility
**MS11**	3.9	40.1	−0.90
**MS12**	5.2	42.6	−0.87
**MS13**	4.45	45.2	−0.88
**MS14**	2.34	48.91	−0.95
**MS21**	398.16	164.03	0.78
**MS22**	324.25	136.45	0.58
**MS23**	270.3	106.2	0.61
**MS24**	360.5	112.6	0.69

## 5. Models of Spring Mound Formation: Implications for Mars

The occurrence of spring mounds within the Sidi El Hani discharge playa and the Chrita saline lake may be compared to the occurrences of putative spring mounds at several locations on the Martian surface, such as those in Arabia Terra [35,41]. The formation of spring mounds both in eastern Tunisia and in some provinces on Mars follows common tectonic and/or hydraulic pathways combined with eolian sedimentation covering the migrating fluids.

### 5.1. Tectonic Model

From a geodynamic point of view, the MCSH system experiences a compressive tectonic process. This tectonic activity is due to the N–S convergence of the Africa and Eurasia plates [38,61,97]. Albeit compressive, this phase results in extensional as well as compressional structures. Similarly, due to thermal cooling, Mars may have experienced a pulse of large-scale global contraction, which originated the contractional structures observed on the surface, such as wrinkle ridges, lobate scarps and thrusts, and reverse faults [110]. Combined with modeling efforts, observations of these structures at the surface provide significant insights on the thermal and geodynamic evolution of Mars subsurface. As it is the case at the MCSH system, compressional phases on Mars have the potentiality to result in extensional structures, which may in turn enhance the formation of fault spring mounds.

**Figure 25:**
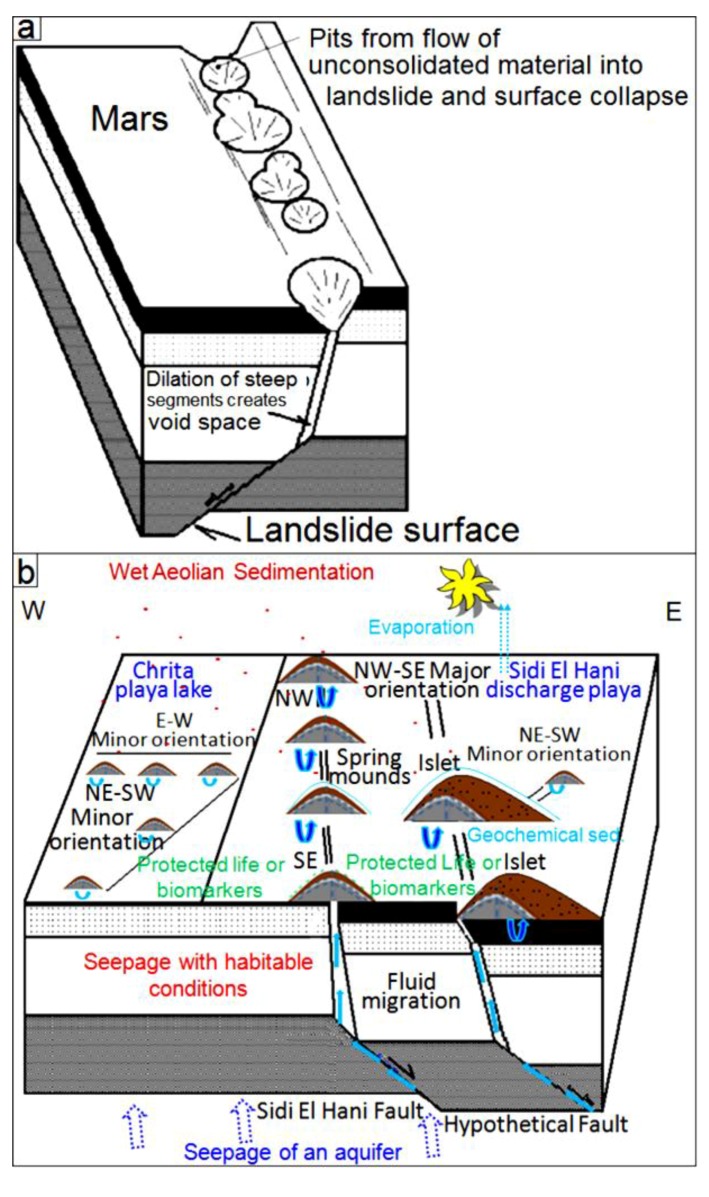
(**a**) Tectonic model of fault spring mounds and mud volcanoes formation on Mars (modified from Kangi [130]); (**b**) Model of the Mechertate-Chrita-Sidi El Hani system: water seepage towards playa surfaces.

All this activity reinforces the notion that tectonic forces were operating during the formation of spring mounds within the system (Figure 25b), as faulted zones would have created perfect paths for seepage through fractured areas. On Mars, though Smith *et al.* [111] suggested that the development of fractures might be insufficient to permit the required outburst rates, these fractures seem enough to guarantee at least fluids seepage. Previous studies (e.g., [112,113,114,115,116,117,118]) suggested that brecciation and faulting of near-surface of fault zone materials are possible mechanisms for development of values of permeability in the range of 10^−22^ to 10^−12^ m^2^. In addition, although processes such as cataclasis (e.g., [119]) and mineralization (e.g., [120,121]) reduce the permeability along fractures, development of fractures may only facilitate water flow (e.g., [122,123,124]), and even deformation bands that are precursor to faults can increase fluid flows in some cases (e.g., [125]). This possibility is reinforced by the fact that dramatic increases in permeability could conceivably have arisen as a result of the dewatering of hydrous salts [126,127] or the melting of large volumes of relict or segregated ice [128,129].

After all the above considerations, we suggest that the tectonic model for the origin of fault spring mounds in eastern Tunisia may be also applied to Mars. Since we can equally find a genetic link between the organization of the fault spring mounds within the discharge playa and the orientation of faults (Figure 25b), the orientation of the tectonic structures in the subsurface of Mars may be inferred following the organization of fault spring mounds on the surface. The location of fault spring mounds on the edge of an islet according to a NW-SE orientation is yet another argument to propose their tectonic origin within this depression.

The block diagrams presented in Figure 25 show the tectonic model of formation of these features on Mars and at the MCSH system. In this model, we consider a tectonic extension, which may have originated these features on Mars (Figure 25a) [130] and terrestrial systems (Figure 25b) (this study). Compressive as well as extensional faulting are both probably able to provide efficient pathways for fluid seepage. Lobate scarps on Mars have been interpreted to be the result of surface-breaking thrust faults (e.g., [131]). The faulting depths of these lobate scarps on Mars were estimated to 30 km (e.g., [132]). It should be noted that, on Mars, fracturing and development of fault systems due to impact cratering (e.g., [133,134]) could particularly be important for increasing permeability and consequent fluid migration along the faults. Rodriguez *et al.* [135], for example, envisioned a complex network of radial and concentric faults of multiple impact craters on Mars, inducing active groundwater storage and movement. This type of fault networks likely facilitates spring mound formation on the surface.

### 5.2. Hydraulic Model

**Figure 26:**
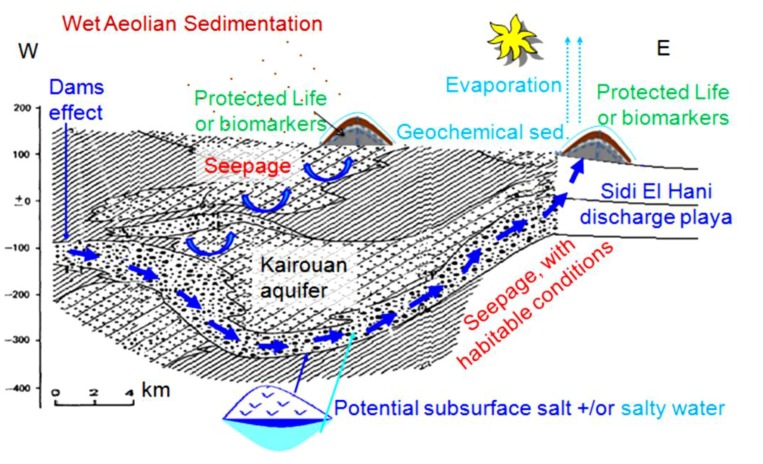
Hydraulic model of spring mound formation in the Mechertate-Chrita-Sidi El Hani system: possible Mars analog (Essefi [38], modified).

The watershed feeds depressions with water and sediments. The Sidi El Hani discharge playa, as the basal part of the system, collects the exceeding water from the Mechertate and Chrita depressions. At the Sidi El Hani discharge playa, being the basal part of the endorheic system, aquifers converge after leaching domes of salt and/or transporting salty water located in the subsurface of the system. Groundwater seepage is another hydrogeological active mechanism in the Tunisian system, also identified to be operating from the Martian subsurface toward its surface [136]. As indicated by many paleo-crater lakes and cataclysmic flood channels studied on the Martian surface (e.g., [14,31,33,137,138,139,140,141,142]), various water-related processes appeared to have operated on the planet, indicating a hydraulic model (Figure 26) as a plausible mechanism for artesian spring mounds formation on Mars. Previous studies showed that similar spring mounds at the Chotts Djerid and Fedjadj in southern Tunisia are fed by point sources of artesian water rising from aquifers in the Continental Intercalaire and Complexe Terminal aquifer series [143].

### 5.3. Hydro-Tectonic Model

**Figure 27:**
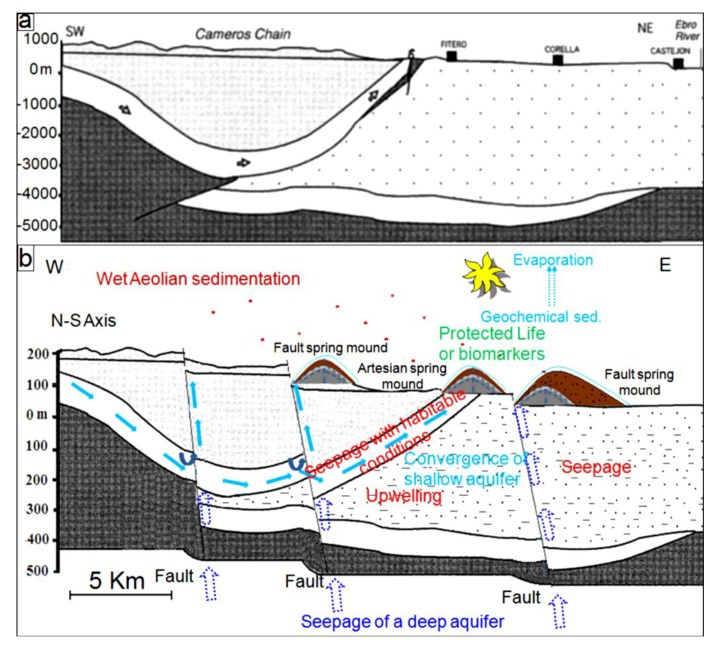
(**a**) Hydro-tectonic model explaining the sedimentary processes related to the groundwater flow from the Mesozoic Carbonate Aquifer of the Iberian Chain in the Tertiary Ebro Basin, northeast Spain (Sánchez *et al.* [144], reinterpreted); (**b**) Hydro-tectonic model of spring mounds formation on Mars and the Mechertate-Chrita-Sidi El Hani system: water upwelling towards playa surfaces.

The tectonic and hydraulic models described here compete to explain the origin of spring mounds in eastern Tunisia and Mars. On one hand, the tectonic model suggests that the faulted subsurface may have originated these features. This model was recently discussed for the Martian case by Kangi [130] (Figure 25a), relating the occurrence of sedimentary mud volcanoes to the internal geodynamics of Mars. On the other hand, other authors advocate the hydraulic model, which may satisfy the emanation of fluids (fresh and/or salty water, CH_4_ and CO_2_) on Mars (e.g., [44]) and groundwater upwelling toward discharge playa surfaces. The co-existence of strong arguments for both models, as we have analyzed and discussed in this paper, allows us to suggest a hydro-tectonic model (Figure 27b), which combines the tectonic and hydraulic scenarios. This hybrid model may offer a consistent explanation of these features both on terrestrial environments and Martian systems.

### 5.4. Eolian Sedimentation

At the MCSH system, once a spring mound is formed due to tectonic and hydrologic conditions, eolian sedimentation dominates the area and promotes the evolution of the spring mound to form an islet (Guattaya). For example, the islet of Ouled Moussa (Figure 4b) shows an increasing accumulation of eolian sediment [38]. Eolian sediments come from Pleistocene to Holocene eolian landforms outcropping in the system [53] or from arid and desert regions located in southern Tunisia. Due to groundwater coming up and salt precipitation, these indurated obstacles further accumulate eolian sediment to set a mixture of eolian and geochemical sedimentation identified as travertine [145] or tufa [146]. Consequently, the sedimentologic investigation of these islets shows their eolian content, which is thicker on bigger islets. In a similar way, we predict eolian sedimentation along an alignment of indurated dunes or mounds on Mars. The induration process involves the presence of ice and/or salt (e.g., [147]). In both cases, groundwater plays a central role in this accumulation.

### 5.5. Inferring Past Hydraulic and Geodynamic Conditions on Mars

**Figure 28:**
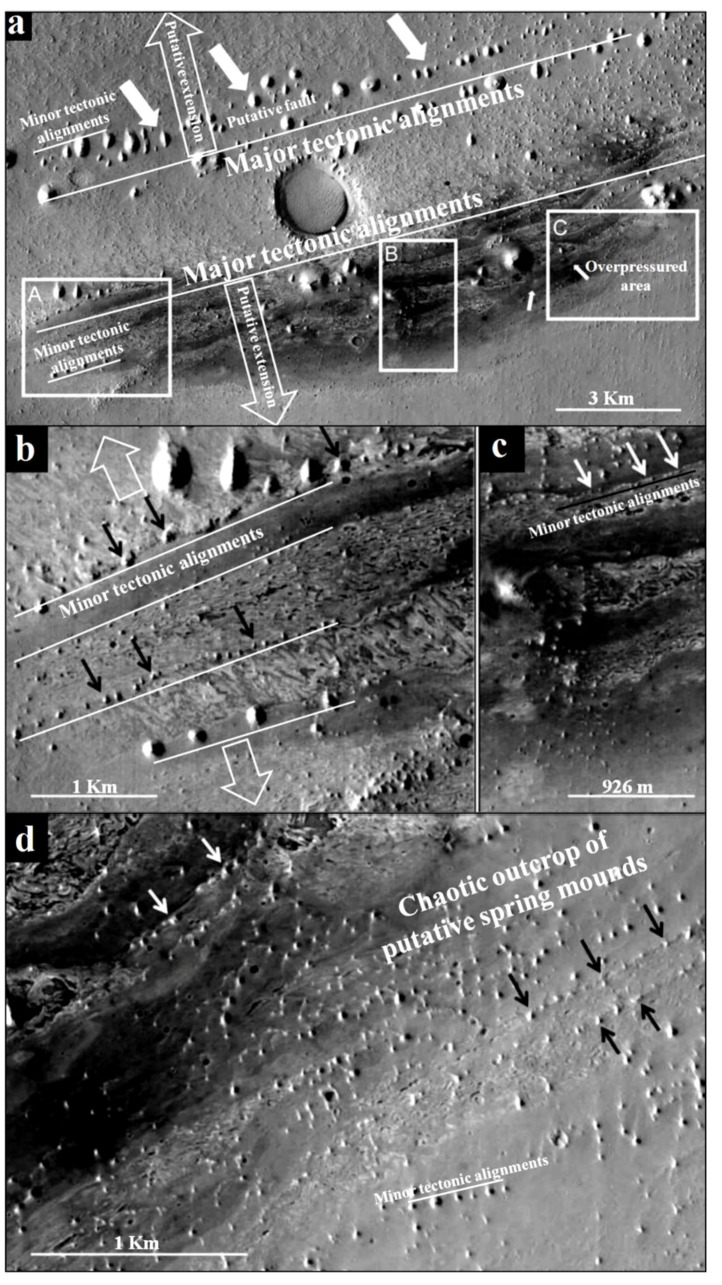
Inference of the geodynamic and hydraulic conditions in the Martian subsurface through spring alignments. Tectonic alignments (**a**–**c**) and chaotic distributions (**d**) of putative spring mounds on Terra Arabia, Mars (after Allen and Oehler [41]).

One of the major aims of this study is inferring the past internal conditions of Mars during the periods of water availability and the putative tectonic activity through the study of its current surface. As shown in Figure 28, Arabia Terra shows putative springs [41] and/or spring mounds [35]. These features may give clues to infer the geodynamic and hydraulic conditions prevailing at the Martian subsurface. For example, the obvious alignment of fault spring mounds along major and minor orientations in Arabia Terra (Figure 28a) is a clear indication of faulting activity, which in turn appears to be due to an extensional phase and/or an effect of global contraction, or reflections of fault system developed due to impact cratering. It is worth mentioning that our study cannot straightforwardly decide whether it is a normal or a reverse fault. However, since our study about these spring mounds in many terrestrial analogs in Tunisia advocates the possibility of normal fault enhancement, we suggest that the extensional hypothesis is the more probable on Mars. On the other hand, the chaotic distribution of spring mounds is likely associated with an overpressured subsurface (Figure 28b).

### 5.6. Magnetic Properties and Remote Sensing Identification of Spring Mounds on Mars

In terrestrial geology, magnetic susceptibility is primarily dominated by iron oxides such as the titanomagnetite series (e.g., [148]). Magnetism on Mars is also believed to be dominated by iron oxides and sulfides (e.g., [149]). Recent Mars exploration argued the surprising detection of strongly magnetized ancient crust on Mars (REFs). Our investigation of spring mounds at the MCSH obviously shows that they are magnetically distinguished from their surroundings. This notice proves vital to distinguish and identify the conversely called spring mounds on Mars. This evidence does not give new findings in terms of instrumentation because the instruments already exist, but it will guide their use. For instance, MIMOS II instrument operating on the Mars Exploration Rover (MER) vehicles is capable of detecting magnetite and hematite at the 1%–2% level [150]. Newer generations of the instrument (e.g., MIMOS-IIa) also show a significant improvement in sensitivity of magnetic susceptibility measurements. Studying these putative spring mounds *in situ* is of intrinsic value to understanding the weathering process, and useful for providing supporting data for interpreting remotely sensed mineralogy.

### 5.7. Implications for Life

Spring mounds on earth (e.g., [16]) and on Mars (e.g., [41,151]) would represent optimal niches of life development. At the MCSH system, depressions contain briny (≈300 g/L) and slightly acid (5.8) water, while springs mounds inject relatively fresh water (7.25 g/L) with neutral pH (6.8). On early Mars, both aqueous systems could have been appropriate for life. First, cold brines with a similar salts concentration to that measured at the MCSH depressions have been proposed to have existed on a “cold and wet” Early Mars [2], potentially adequate for biological development [152]. Second, fresher water associated with springs might have not been as briny or acidic as water in terrestrial evaporating pools [41], and this may have provided a long-term habitable environment on a “warm and wet” early Mars [153]. If life ever developed on Mars, ancient spring deposits would be excellent localities in which to search for morphological or chemical remnants of that life [41], with proper drilling into the accumulated materials [154,155]. These favorable conditions for life, which are exceptionally shown at the surface through spring mounds, may have been more frequent in the Martian subsurface, indicating that geodynamic and hydraulic conditions within the Martian subsurface could have been favorable for biological development.

The mechanisms (tectonic and/or hydraulic) of formation and evolution of spring mounds at the MCSH system are suitable for the proliferation and protection of life respectively. On the one hand, their formation through the upwelling of water [40] and organic-rich material [109] provides with the necessary elements for life development. On the other hand, the protecting layers formed due to the wet eolian sedimentation provide with a safe site for the protected life or remains of the life (e.g., biomarkers). Similarly, life or biomarkers on Mars may have been protected or preserved under the spring mounds.

## 6. Conclusions

We have analyzed here the characteristics of the spring mounds distributed along the Mechertate-Chrita-Sidi El Hani (MCSH) system, eastern Tunisia. We propose that the genesis of these *child* spring mounds is directly caused by groundwater coming up, which is due to hydraulic (artesian spring mound) overpressure and/or tectonic fractures (fault spring mound). Then, eolian deposition intervenes with the water table to control their evolution towards distinctive *islets* spring mounds. This hypothesized sequence is supported by the fact that the internal core of the spring mounds consists of endogenous mud mixed with groundwater, whereas the external wrap is covered by eolian deposition enhanced by the water table. Some of these spring mounds are in continuous activity due to a constant supply of salty water. Others are in a seeping stage, because water pressure is not enough to induce aqueous upwelling and therefore water seeps laterally and causes the deposition of a travertine at the mouth of the spring. These artesian springs appear to be the result of the hydraulic pressure generated by the convergence of aquifers towards the surface of the system. Therefore, a hydraulic model ought to be considered in the analysis of the formation of the spring mounds. However, the fault spring mounds are organized along preferential orientations probably inherited from current or past tectonic activity. This observation advocates for a tectonic model of spring mounds formation. Thus, both models merit consideration to fully understand the formation and evolution of the spring mounds at the MCSH system. We propose here to adopt a combined hydro-tectonic model to describe Tunisian spring mounds, a model that can also be useful for the analysis of Mars’ purported spring mounds. The study presented here of the MCSH terrestrial analog may be valuable to test different models of Mars’ spring mound origin. The Martian subsurface may be similarly over-pressured and fractured, and therefore our combined hydro-tectonic model may be adequate to describe the formation and evolution of spring mounds on Mars. Spring mounds are safe zones for the setting and development of life. The feeding from the subsurface ensures the coming up of water and organic-rich material. The wet eolian sedimentology covers the safe site protecting life or biomarkers.
